# Using Genome-Wide Association Analysis to Characterize Environmental Sensitivity of Milk Traits in Dairy Cattle

**DOI:** 10.1534/g3.113.006536

**Published:** 2013-07-01

**Authors:** Melanie Streit, Robin Wellmann, Friedrich Reinhardt, Georg Thaller, Hans-Peter Piepho, Jörn Bennewitz

**Affiliations:** * Institute of Animal Husbandry and Breeding, University of Hohenheim, 70593 Stuttgart, Germany; † Vereinigte Informationssysteme Tierhaltung w.V. (VIT), 27283 Verden, Germany; ‡ Institute of Animal Breeding and Husbandry, Christian-Albrechts-University, 24098 Kiel, Germany; § Institute of Crop Science, University of Hohenheim, 70593 Stuttgart, Germany

**Keywords:** environmental sensitivity, genotype-by-environment interaction, association analysis, dairy cattle

## Abstract

Genotype-by-environment interaction (GxE) has been widely reported in dairy cattle. One way to analyze GxE is to apply reaction norm models. The first derivative of a reaction norm is the environmental sensitivity (ES). In the present study we conducted a large-scale, genome-wide association analysis to identify single-nucleotide polymorphisms (SNPs) that affect general production (GP) and ES of milk traits in the German Holstein population. Sire estimates for GP and for ES were calculated from approximately 13 million daughter records by the use of linear reaction norm models. The daughters were offspring from 2297 sires. Sires were genotyped for 54k SNPs. The environment was defined as the average milk energy yield performance of the herds at the time during which the daughter observations were recorded. The sire estimates were used as observations in a genome-wide association analysis, using 1797 sires. Significant SNPs were confirmed in an independent validation set (500 sires of the same population). To separate GxE scaling and other GxE effects, the observations were log-transformed in some analyses. Results from the reaction norm model revealed GxE effects. Numerous significant SNPs were validated for both GP and ES. Many SNPs that affect GP also affect ES. We showed that ES of milk traits is a typical quantitative trait, genetically controlled by many genes with small effects and few genes with larger effect. A log-transformation of the observation resulted in a reduced number of validated SNPs for ES, pointing to genes that not only caused scaling GxE effects. The results will have implications for breeding for robustness in dairy cattle.

Breeding cattle for milking traits relies on the use of daughter records for an estimation of breeding values of their sires. Because sires are used widely through artificial insemination, their breeding values are estimable with a high accuracy, which has resulted in a substantial genetic gain for milking traits during the last decades (Dekkers and Hospital 2002). It is expected that this gain will be even further accelerated with the introduction of genomic selection methods (Meuwissen *et al.* 2001; Goddard and Hayes 2009). Often, frequently used sires have daughters that are milked in a wide range of environments, which questions the importance of genotype-by-environment interaction (GxE). GxE refers to a variable response of genotypes to changes in the environment. Many studies have been conducted to quantify putative GxE effects in dairy cattle (*e.g.*, Kolmodin *et al.* 2002; König *et al.* 2005; Strandberg *et al.* 2009 and references therein). The use of reaction norms is a powerful approach to study GxE effects if the environment can be described as a continuous variable. The slope of a reaction norm, *i.e.*, the first derivative, is the environmental sensitivity (ES), and genetic variation of ES can be interpreted as the existence of GxE (de Jong 1995; Lynch and Walsh 1998; James 2009).

A frequently used environmental descriptor is the mean performance of all individuals in the environment (James 2009). It is assumed that various, unknown, or unobservable environmental forces affect the mean performance. Mean performance is therefore a descriptor that captures these effects and weights them in a “natural” way, *i.e.*, by their effects on the performance. In dairy cattle, reaction norm models that include the average herd production level as a continuous environmental descriptor are widely used to study GxE (Calus *et al.* 2002; Kolmodin *et al.* 2002; Fikse *et al.* 2003; Hayes *et al.* 2003; Strandberg *et al.* 2009; Lillehammer *et al.* 2009a; Streit *et al.* 2012). Reaction norms frequently are fitted with the use of random regression sire models. The daughter’s observations are regressed on the corresponding herd solution. The regression is nested within sires, yielding a random sire estimate for the slope and for the intercept. The correlation between intercept and slope depends on where the intersection point of the reaction norm model is placed; it is recommended that it be placed in the average environment (van Tienderen and Koelewijn 1994; Kolmodin and Bijma 2004). In this case the intercept estimate can be interpreted as an estimate for average or general production (GP) and the slope as an estimate for ES for individual sires. A positive correlation between intercept and slope under this conditions frequently has been reported (*e.g.*, Kolmodin *et al.* 2002, Lillehammer *et al.* 2009b).

It might be worthwhile to consider ES in livestock breeding schemes (de Jong and Bijma 2002, Knap 2005, Veerkamp *et al.* 2009). Breeding for high yielding and sensitive individuals might be beneficial in high-producing and nonfluctuating environments because sensitive individuals are able to benefit from these environmental conditions. In poor, fluctuating, or unforeseeable environments, robust individuals are desired, if the robustness does not come at the expense of a decrease in fitness and increase in health problems. One way to breed simultaneously for robustness and GP is to find genes that affect GP and ES of one trait in opposite directions and to apply marker-assisted selection using these genes (Lillehammer *et al.* 2009b). Lillehammer *et al.* (2009b) applied association analysis by using approximately 10,000 single-nucleotide polymorphisms (SNPs) in the Australian dairy cattle population to find significant SNPs affecting GP and ES. Several SNPs were significant, and approximately one-third affected GP and ES in opposite directions; these SNPs are of special interest with regards to breeding for robustness.

The genetic architecture of dairy cattle milk traits been has analyzed frequently(*e.g.*, Cole *et al.* 2009, Hayes *et al.* 2010, Wellmann and Bennewitz 2011). However, it is unknown how many genes affect ES, what the sizes and distributions of the effects are, and where they are located on the genome. In a recent study, we applied higher-order reaction norm random regression sire models to investigate GxE effects in German Holsteins (Streit *et al.* 2012). Herd test day solutions for production were used as environmental descriptors. We found highly significant GxE for milk traits, which resulted in substantial scaling and few re-ranking effects. For a deeper understanding of the nature of GxE effects, a partitioning of GxE effects into that due to scaling and due to changes in the rank of individuals across environments is desirable (*e.g.*, Muir *et al.* 1992, Dutilleul and Potvin 1995, James 2009). An obvious method to reduce or eliminate scaling effects is to apply a data transformation (James 2009), which would allow partitioning of removable by data transformation and nonremovable interaction.

The aim of the present study was to conduct a validated genome-wide association analysis to identify SNPs that affect GP and ES, and based on the results, to infer some knowledge of the genetic architecture of GP and ES. We were especially interested in the number of validated SNPs and the size and the sign of the effects on GP and ES. We applied a three-step procedure. In the first step, sire estimates for GP and for ES were calculated by the use of first-order random regression sire models. These estimates were used in a second step as observations in an association analysis. In the third step, significant SNP associations were confirmed in an independent validation set of the same population. To remove GxE causing scaling effects, the observations were log-transformed in some analyses.

## Materials and Methods

### Data and data editing

In total 2356 progeny-tested German Holstein sires were genotyped with the Illumina BovineSNP50 BeadChip, which contains a total of 54,001 SNPs (Illumina, San Diego, CA; Matukumalli *et al.* 2009). The sires were born between 1983 and 2003 and reflect a representative sample of the population (Qanbari *et al.* 2010). Individuals with more than 10% missing marker genotypes were removed, resulting in 2297 sires. An SNP was excluded if it had a minor allele frequency less than 3%, a call rate less than 90%, a significant deviation from the Hardy-Weinberg-equilibrium (*P* < 0.001), or if the position on the genome was unknown. SNPs on the sex chromosome also were excluded. This data filtering was performed with PLINK (Purcell *et al.* 2007). A total of 41,349 SNPs remained in the data set. Sporadic missing genotypes were imputed using fastPHASE (Scheet and Stephens 2006). The linkage disequilibrium (LD) structure in this population was investigated by Qanbari *et al.* (2010).

Approximately 13 million first lactation test day records for protein yield, fat yield, and milk yield from daughters of the sires were used. The number of daughters per bull ranged from 50 to 74,842 and totaled approximately 1.3 million. Test day records were corrected for the fixed effects herd test day, days in milk, age at calving, calving season, and the random permanent environment effect. These correction factors were obtained from the routine animal genetic evaluation, which is an animal test day model. After this adjustment, the trait population mean was added to the observations to obtain predicted trait values.

The environment was described by the mean herd test day performance for milk energy yield. It was calculated as a linear combination of milk yield, fat yield and protein yield, *i.e.*, *energy yield* = 0.802 x *milk yield* + 38.4 x *fat yield* + 23.6 x *protein yield*, where the yields are measured in kilograms (Nostitz and Mielke 1995). We preferred this single parameter to describe the environment because it combined the highly correlated herd test day performances for the three milk yield traits (see Streit *et al.* 2012 for further details). It is assumed that this parameter captures important unobservable and unknown environmental effects. The environmental descriptor was rescaled to have a mean equal to 0 and SD of 1. Hence, superior (inferior) environments show positive (negative) values for the environmental descriptor, and the “average” environment shows a value close to zero. The distribution of the environmental descriptor is shown in the Supporting Information, Figure S1. It is approximately normally distributed. Mean herd test day performances of milk yield, protein yield, and fat yield were obtained from the routine animal genetic evaluation, see Vereinigte Informationssysteme Tierhaltung (2013) for a detailed description. For additional information please see File S2, File S3, and File S4.

### Statistical analysis

In a previous study we applied a second-order sire model, which gave an improved fit compared with a first-order model. However, a first-order sire effect explained most of the variation of ES (Streit *et al.* 2012). Therefore, we decided to apply a fist-order sire model in the present study. The following random regression model was applied in the first step for all three milk yield traits:

$$cy_{ijk}=\mu +b^{*}htdsme_{k}+\sum_{m=0}^{1}{s_{jm}}^{*}htdsme_{k}^{m}+\sum_{m=0}^{1}{d_{ijm}}^{*}htdsme_{k}^{m}+e_{ijk},$$ (1)

where *cy_ijk_* is the corrected yield of daughter *i* of sire *j* at herd test day *k*, *µ* is the overall mean, *htdsme_k_* is the herd test day solution for milk energy yield at herd test day *k* with the fixed regression coefficient *b*, *s_jm_* is the random sire effect of sire *j* of order *m*, *d_ijm_* the random daughter effect of daughter *i* of sire *j* of order *m*, and *e* is the random residual. The covariance structure of the sire regression effects is $Var\left[\begin{array}{l} s_{0}\\ s_{1} \end{array}\right]=I⊗[\begin{array}{l} \sigma_{s_{0}}^{2}\\ \sigma_{s_{0}s_{1}} \end{array}\begin{array}{l} \sigma_{s_{0}s_{1}}\\ \sigma_{s_{1}}^{2} \end{array}]$, and that of the daughter effects is $Var\left[\begin{array}{l} d_{0}\\ d_{1} \end{array}\right]=I⊗[\begin{array}{l} \sigma_{d_{0}}^{2}\\ \sigma_{d_{0}d_{1}} \end{array}\begin{array}{l} \sigma_{d_{0}d_{1}}\\ \sigma_{d_{1}}^{2} \end{array}]$, with *I* being the identity matrix. The estimated sire effects were used as observations in an association analysis [see model (2)]. In contrast to classical sire models, the relationship among sires was ignored, which could be done because there was much progeny information available for each sire, and hence, the sire estimates were largely influenced by the progeny records and only very little by the pedigree. Indeed, preliminary results showed that the correlation between sire estimates with and without considering the pedigree in model (1) was >0.98 (not shown).

To model heterogeneous residual variance across the environments, the observations were ordered according to the environmental descriptor and grouped into 10 classes of equal size based on the environmental values. Residual variances were estimated for each class, assuming the residual covariance to be zero. The uncorrelated daughter effects reduce the heterogeneity of residual variance if GxE effects are present (Lillehammer *et al.* 2009a). The models were fitted using ASReml 3.0 (Gilmour *et al.* 2009). Because the mean of the environmental descriptor was zero, the intercept solutions of the sire regression coefficients were used as sire estimates for GP, *i.e.*, production level in the average environment. Furthermore, the slope solutions were used as estimates for ES.

The whole data set was randomly split into a discovery data set (*n* = 1797 bulls) and a validation data set (*n* = 500 bulls). In the second step of the statistical analysis, we performed genome-wide association analyses using the discovery data set. To do so, we applied the following mixed linear model:

$${\hat{s}}_{jm}=\mu_{m}+sire_{jm}+{b_{km}}^{*}x_{jk}+e_{jm}$$, (2)

where ${\hat{s}}_{jm}$ is the estimated random sire effect for GP (*m* = 0) and ES (*m* = 1). These estimates were taken from the results of model (1). The model was applied for the two traits (*m* = 0 for GP and *m* = 1 for ES) separately. The effect of the SNP *k* was modeled as a regression on the number of copies of the allele with the greater frequency (*x* = 0, 1, or 2), with $b_{km}$ being the regression coefficient. To control for the population structure, we fitted a random sire effect with the covariance structure$A\sigma_{sm}^{2}$, where *A* is the numerator relationship matrix calculated from high-quality pedigree information and $\sigma_{sm}^{2}$ a variance attributable to the sires. This model was applied for each SNP *k* in turn, resulting in 41,349 association tests per trait. We declared each SNP with a pointwise error probability of *P* < 0.001 as significant. To judge how many false-positive results were among the significant associations we applied the false-discovery rate (FDR) technique. We calculated for each association test an FDR *q*-value by using the software QVALUE (Storey and Tibshirani 2003). The FDR *q*-value of the significant SNP with the lowest test statistic (*P* ≈ 0.001) provided an estimate of the proportion of false-positive results among the significant associations.

In the third step, we confirmed significant SNP associations within the same population in the validation set. The same statistical model was applied but only to significant SNPs. We declared an SNP as confirmed if the *P*-value in the validation set was *P* < 0.01 and the signs of the effects were the same in both sets. This relaxed significance criterion was used in the validation set because less multiple testing was performed and a more stringent significance level would reduce the power to validate SNPs. A similar protocol was used by Pryce *et al.* (2010). For the interpretation of the effects, the estimates of the validation set were used, because it can be assumed that these suffer less from the Beavis-effect and are less upwardly biased (Beavis 1994).

To identify SNPs that not only cause scaling effects within the environmental range considered in our study, we also applied the models to log-transformed observations (Hayes *et al.* 2003, Lillehammer *et al.* 2009b). Preliminary results revealed convergence problems of model (1) with log-transformed observations (not shown), which was caused by the random regression of the daughter on the environment. Therefore, to ensure convergence, the random daughter effect was fitted without regression on the environment. The residual variance was homogeneous, so only one residual variance component was estimated. The sire solutions obtained from model (1) were used subsequently in model (2).

## Results

The main results of the variance component estimation are shown in Table 1. There is slope variance for all traits on both the observed and the log-transformed scales, pointing to the presence of GxE effects. These GxE effects were analyzed in details and also tested for significance in an earlier study (Streit *et al.* 2012). On the observed scale, the correlation between intercept and slope was high and positive. The log-transformation reduced this correlation. As expected, the daughter variance was substantial and the residual variance was heterogeneous across the environmental classes for traits on the observed scale (not shown).

**Table 1:** Sire variance components of the random regression analyses

Trait	$\sigma_{S_{0}}^{2}$	$\sigma_{S_{1}}^{2}$	$\rho_{S_{0}S_{1}}$
Protein yield, g	2379.37 (87.48)	17.02 (0.98)	0.79
Fat yield, g	7883.41 (257.12)	46.76 (2.43)	0.93
Milk yield, kg	1.30 (0.04)	0.02 (< 0.01)	0.72
ln(protein yield)*^a^*	9.50 (< 0.01)	0.11 (< 0.01)	0.61
ln(fat yield)*^a^*	12.70 (< 0.01)	0.13 (< 0.01)	0.73
ln(milk yield)*^a^*	10.55 (< 0.01)	0.14 (< 0.01)	0.68

Standard errors are shown in parentheses. $\sigma_{S_{0}}^{2}$ ($\sigma_{S_{1}}^{2}$) denotes the intercept (slope) sire variance, with correlation$\rho_{S_{0}S_{1}}$.

a Values are multiplied by 10,000.

The results of the association analysis for the traits on the observed scale are shown in Table 2. For all traits, 350 to 450 SNPs showed a nominal significant association in the discovery data set; the FDR-analysis revealed that approximately 7–9% of these are false-positive results. For fat and protein yield, more trait-associated SNPs could be found for intercept than for slope. The number of validated SNPs was between 44 (protein slope) and 118 (fat intercept). The results for the log-transformed data sets are shown in Table 3. For the intercepts, almost the same number of significant SNPs was found as on the observed scale, but fewer could be confirmed. For the slopes, the number of significant SNPs was reduced. The FDR *q*-values of the significant associations were similar or slightly greater.

**Table 2:** Number of discovered and validated SNPs for intercept and slope for the traits on the observed scale

Trait	Discovery Dataset (*P* ≤ 0.001)	FDR	Validation Dataset (*P* ≤ 0.01)
Intercept protein yield	450	0.07	69
Slope protein yield	351	0.09	44
Intercept fat yield	465	0.07	118
Slope fat yield	385	0.08	99
Intercept milk yield	415	0.08	104
Slope milk yield	416	0.08	98

The FDR *q*-values (FDR) of the significant SNP with the largest error probability (*P* ≈ 0.001) in the discovery dataset are shown. SNP, single-nucleotide polymorphism; FDR, false-discovery rate.

**Table 3:** Number of discovered and validated SNPs for intercept and slope for the traits on the log-scale

Trait	Discovery Dataset (*P* ≤ 0.001)	FDR	Validation Dataset (*P* ≤ 0.01)
Intercept ln(protein yield)	463	0.07	56
Slope ln(protein yield)	313	0.11	64
Intercept ln(fat yield)	469	0.07	118
Slope ln(fat yield)	320	0.11	80
Intercept ln(milk yield)	419	0.08	87
Slope ln(milk yield)	386	0.09	68

The FDR *q*-values (FDR) of the significant SNP with the largest error probability (*p*≈0.001) in the discovery dataset are shown. SNP, single-nucleotide polymorphism; FDR, false-discovery rate.

**Figure 1:**
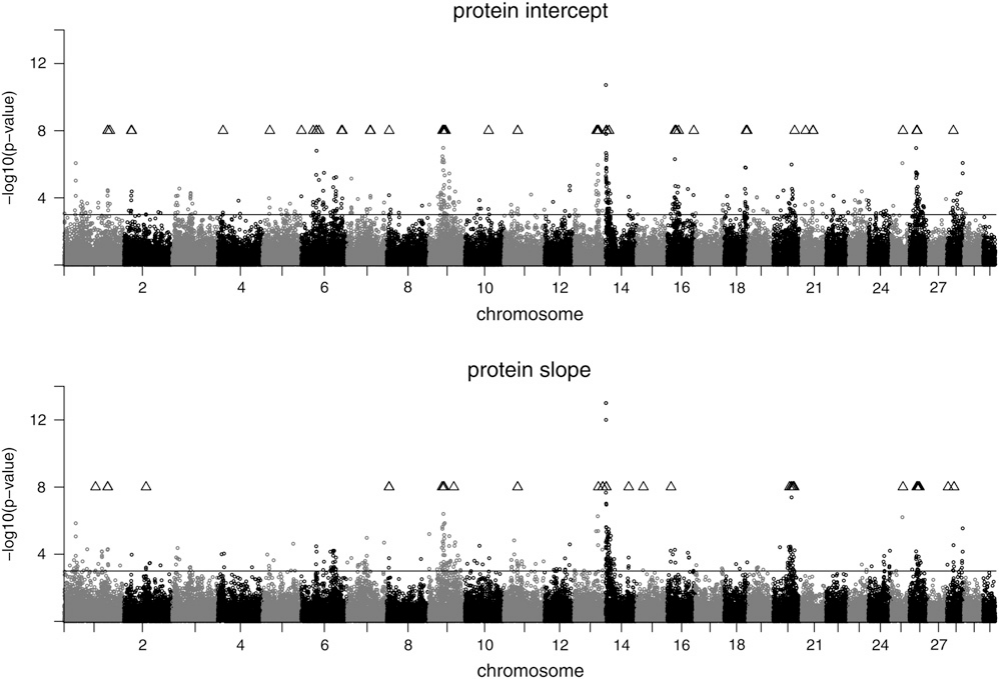
Test statistic profile of SNP effects for protein yield intercept (top) and protein yield slope (bottom) in the discovery data set. The nominal significance level (*P* < 0.001) is indicated by a solid line. Positions of validated SNPs are indicated by a triangle.

**Figure 2:**
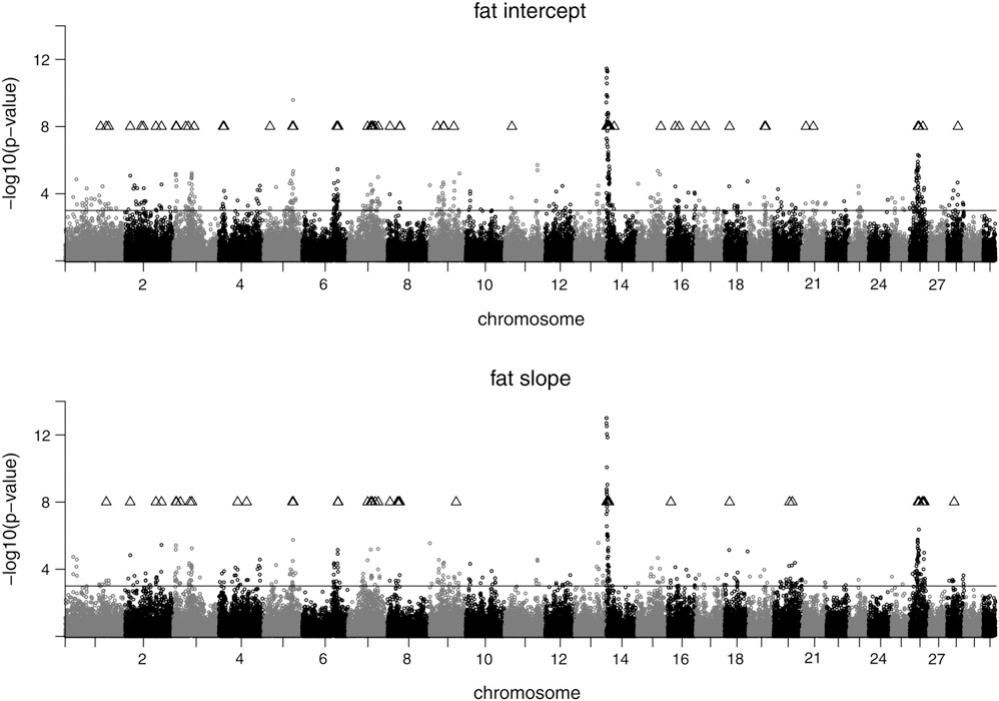
Test statistic profile of SNP effects for fat yield intercept (top) and fat yield slope (bottom) in the discovery data set. The nominal significance level (*P* < 0.001) is indicated by a solid line. Positions of validated SNPs are indicated by a triangle.

**Figure 3:**
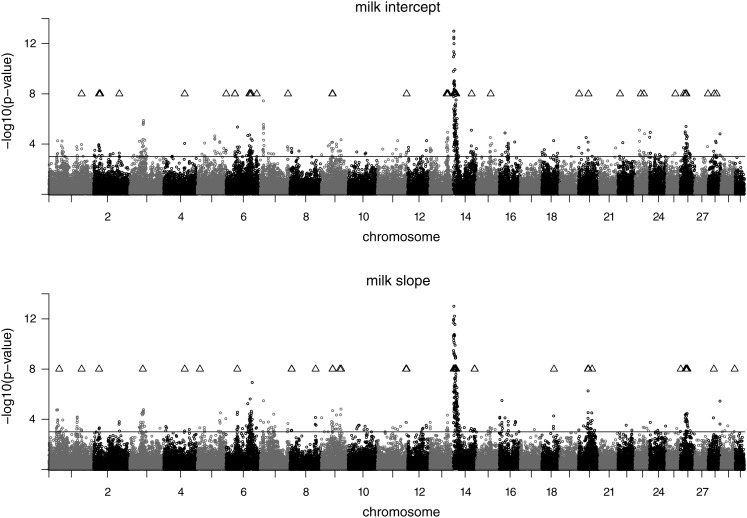
Test statistic profile of SNP effects for milk yield intercept (top) and milk yield slope (bottom) in the discovery data set. The nominal significance level (*P* < 0.001) is indicated by a solid line. Positions of validated SNPs are indicated by a triangle.

**Figure 4:**
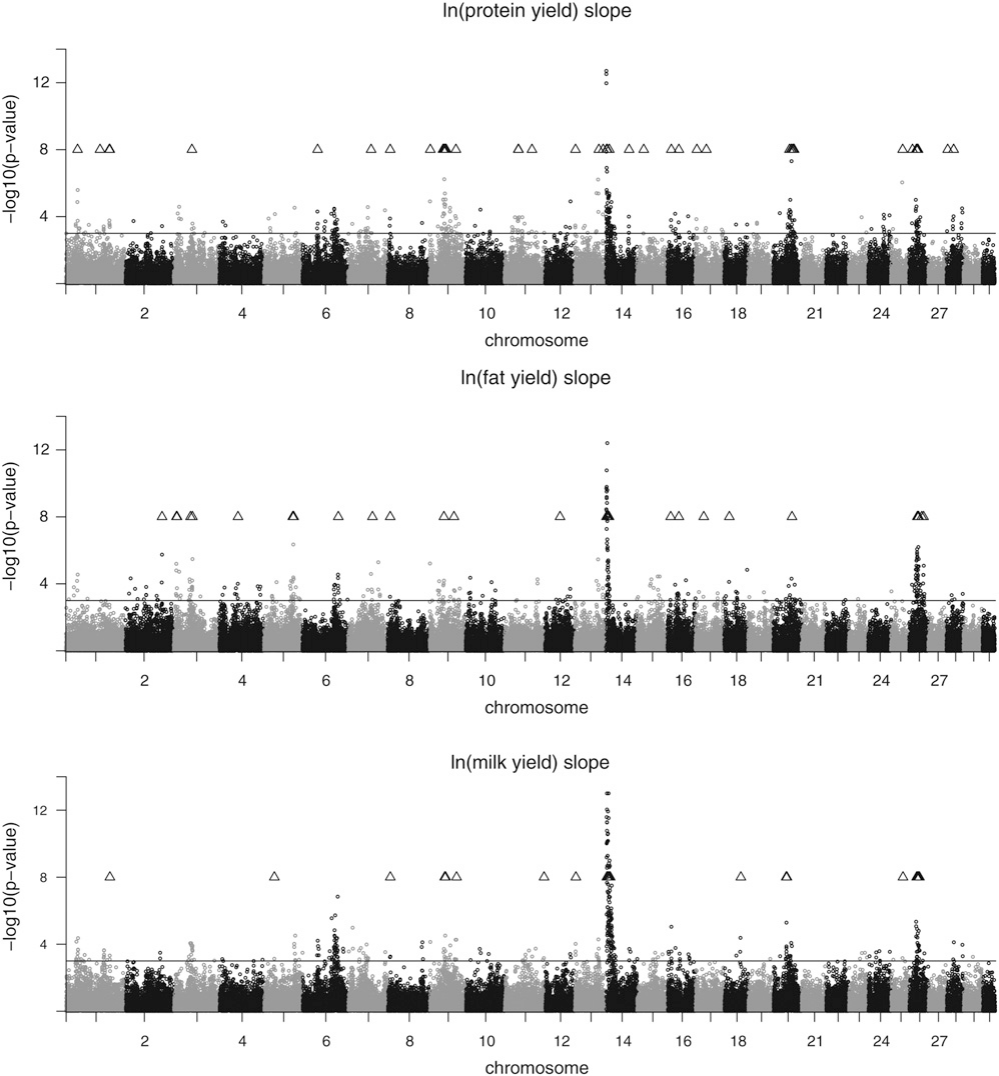
Test statistic profile of SNP effects for ln(protein yield) slope (top), ln(fat yield) slope (middle), and ln(milk yield) slope (bottom) in the discovery data set. The nominal significance level (*P* < 0.001) is indicated by a solid line. Positions of validated SNPs are indicated by a triangle.

The plots of the test statistic along the chromosomes are shown in Figure 1, Figure 2, and Figure 3 for the traits on the observed scale. Chromosomal positions of validated SNPs are indicated by a triangle symbol. The pattern of the test statistic was similar for the intercept and slope within the traits, although for intercept the signals were generally more pronounced, leading to the higher number of significant associations. Significant SNPs were found on many chromosomes, and the clearest signals were observed on BTA14. Promising SNP clusters affecting intercept and slope of all traits were also identified on BTA26. Chromosome 9 is interesting with regard to protein, as it contains a validated SNP cluster for both intercept and slope. For slope, validated SNPs with a remarkably high test statistic were found on BTA20 and BTA25. For fat intercept, a highly significant SNP was found on BTA5, which also affected slope to a lesser extent. For milk slope validated SNPs were mapped on BTA6 and BTA20. The test statistic plots for intercept on the observed and on the log-scale are almost identical for all three traits (not shown). For slope, however, the plots differ between the scales (see Figure 4). Again, SNPs on BTA14 showed the strongest signals for all three log-transformed traits for slope.

**Figure 5:**
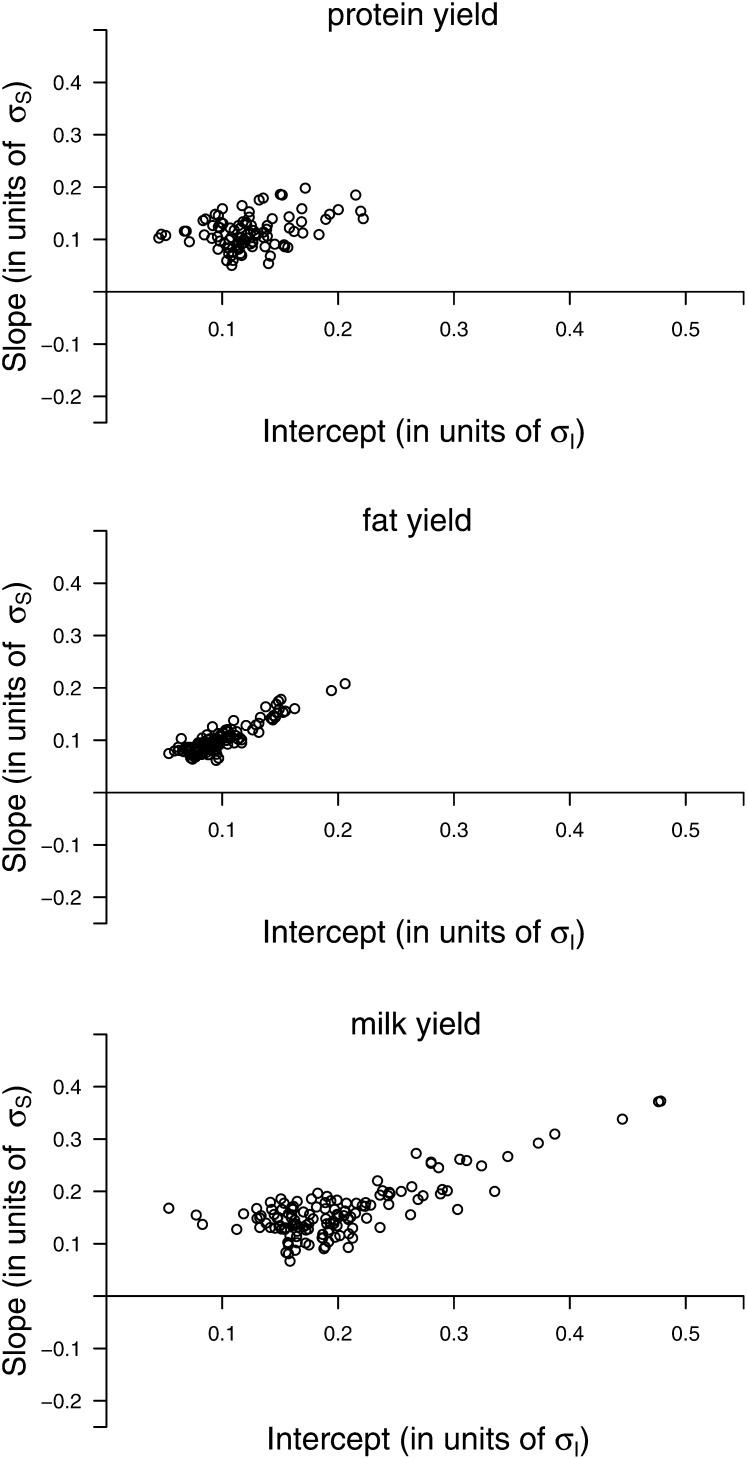
Estimated SNP effects for the traits on the observed scale. The term $\sigma_{S}$ ($\sigma_{i}$) denotes the sire intercept (slope) SD. Each SNP was validated within the population either for intercept, slope or both. Estimates were taken from the validation set.

**Figure 6:**
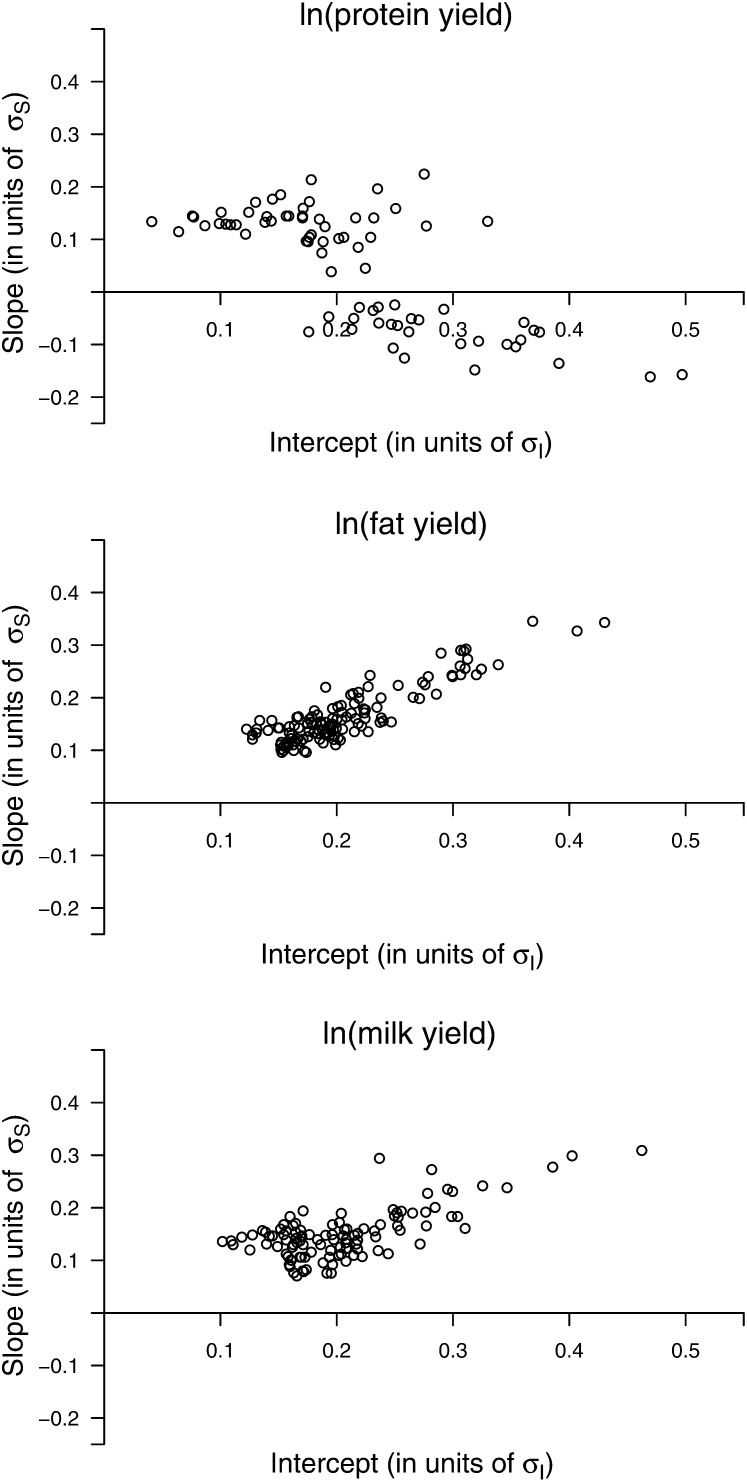
Estimated SNP effects for the traits on the log-scale. The term $\sigma_{S}$ ($\sigma_{i}$) denotes the sire intercept (slope) SD. Each SNP was validated within the population either for intercept, slope or both. Estimates were taken from the validation set.

In Figure 5 the estimates of the validation set are shown for SNPs that were either significant for intercept, or for slope, or for both. The slope effect of the allele that increases the intercept is shown. It can be seen that every validated SNP affects both traits in the same direction, and the correlation between the solutions is highly positive. This was less pronounced if the data were log-transformed (Figure 6). For ln(protein yield), many validated SNPs for intercept showed a small but mostly non-significant negative effect for slope. In general, the largest SNP effects (in units of the standard deviation, σ) were observed for milk yield, with 11 (4) SNPs showing an intercept (slope) effect larger than 0.3σ. For the log-transformed data sets, the intercept effects are generally larger. This was not observed for slope effects. The estimates of each validated SNP for the traits on the observed scale are presented in Table S1; estimates for the log-transformed observations are presented in Table S2.

## Discussion

In this study we attempted to identify and confirm SNPs for intercept (reflecting GP) and slope (reflecting ES) of milk traits in the German Holstein dairy cattle population. Numerous SNPs were identified and confirmed for both GP and ES. Many SNPs affecting GP also affect ES. We showed that ES of milk traits has a similar genetic architecture as GP and is a typical quantitative trait, genetically controlled by many genes with small effects and few genes with larger effect (Figure 5, Table S1). Given the FDR *q*-values of the SNPs in the discovery set (Table 2 and Table 3), it seems that some SNPs with true associations were not confirmed. This might be due to the reduced power of the validation set with 500 sires. A more stringent validation would be to also test whether the SNP is significant in another population (Hayes *et al.* 2009, Pryce *et al.* 2010). Such a validation study would also increase mapping precision, because mapping resolution is increased when using an across-breed approach and only those SNPs being in LD with the mutation in both breeds would be validated. No independent population was available, however, to do an across-population validation in this study.

In our study, the mapping precision is limited due to the LD structure observed in this population (Qanbari *et al.* 2010) in combination with the applied single marker association analysis. Alternatively, a combined linkage and LD mapping approach could have been applied, which predicts IBD-probabilities at putative quantitative trait loci (QTL) regions using multimarker and pedigree information and uses these probabilities for QTL fine-mapping (Meuwissen *et al.* 2002). This method is, however, computationally demanding and needs greater marker densities. Another multimarker approach that could have been applied is a Bayes-method originally developed for genomic selection (Meuwissen *et al.* 2001, Goddard and Hayes 2009). These Bayes-methods make use of the LD of the markers and the mutation and additionally of the LD between the markers. It is not completely clear how to test for significance when using these methods. Olsen *et al.* (2011) applied the three aforementioned approaches to map genes for fertility and milk production in dairy cattle. They applied single-marker association analysis for a first screen, fine-mapped the regions by using combined linkage and LD mapping, and confirmed the putative positions by using BayesA from Meuwissen *et al.* (2001).

Some interesting SNP clusters affecting GP are located closely to well-known candidate genes that segregate in the German Holstein population. This is most obvious on BTA14, were the clear signals for all milk traits for GP and ES probably reflect the effect of *DGAT1* (Grisart *et al.* 2002, Winter *et al.* 2002). This gene is known to segregate and affect all milk traits in this population (Bennewitz *et al.* 2004a). Several SNPs affecting GP of all three investigated milk traits were found on BTA6. From previous linkage analyses, it is known that BTA6 harbors QTL affecting milk traits in this population (Kühn *et al.* 1999, Bennewitz *et al.* 2004b). Putative candidate genes underlying mapped QTL are discussed in Weikard *et al.* (2005). The *PPARGC1A* gene was postulated as the most plausible gene underlying a QTL for fat yield. In addition, the casein gene complex is located on this chromosome, with an effect on protein yield and protein percentage traits in this population (Prinzenberg *et al.* 2003). On BTA5 we found a single SNP with a remarkably high test statistic for fat GP, which was also validated for fat ES. Wang *et al.* (2012) reported the gene *EPS8* to be most likely causative for this association. The significant SNPs on BTA20 is very likely to be associated with the *GHR* gene (Blott *et al.* 2003, Wang *et al.* 2012).

Some validated SNPs for ES are in chromosomal regions similar to those found in other dairy cattle populations harboring genes with GxE effects. In the Norwegian Red, milk production QTL for ES on BTA2, BTA6, BTA7, and BTA16 were reported by Lillehammer *et al.* (2007, 2008). A detailed analysis of BTA6 with a high marker density revealed two QTL for milk yield with an effect on ES, but no QTL with an ES effect for fat and protein yield. In our population, we were able to validate ES SNPs on BTA6 for milk and fat yield,but not for protein yield. In the Australian Holstein population Lillehammer *et al.* (2009b) found several SNPs with ES effects. Roughly one third of their significant associations affected GP and ES in opposite directions, which is in contrast to our findings. They stated, however, that this proportion is probably smaller than one third because it is generally more difficult to find SNPs that affect GP and ES in the same direction rather than in opposite directions. Our study is considerably more powerful than that of Lillehammer *et al.* (2009b); hence, we were likely able to detect more SNPs with effects in the same direction.

We previously reported significant GxE resulting in substantial scaling effects (Streit *et al.* 2012). To remove these scaling effects, a log-transformation was applied. The results from the association analysis applied to the log-transformed data revealed SNP that were not removable by this kind of transformation. These validated SNPs are of special interest because they point to chromosomal regions harboring genes with an effect on ES that are not or not solely due to scaling effects. Some regions with clear signals for ES on the observed scale could not be found on the log-scale. This was especially observed for ln(protein yield) and SNPs on BTA14 close to the *DGAT1* gene, where positive effects on ES were turned into small negative effects, although mostly not significant (Figure 6, Table S2). Hence, these effects were completely removable by the log-transformation. It may be noted that the log-transformation is frequently applied, but maybe another transformation function (*e.g.*, from the Box-Cox-family of transformation) would be able to eliminate scaling effects more effectively. This was not investigated further in this study. The reduced correlation between intercept and slope when applying the log-transformation (Table 1) was also observed by Lillehammer *et al.* (2009b). This decreased correlation has the following reason. For large yields the intercept of a regression is large as well. Because the logarithm is a concave function, the interval containing these yields is mapped to a smaller interval than an interval of the same size containing small yields. Thus, the transformation causes large yields to decrease in variance more drastically than small yields. This causes positive slopes of the regression lines for large yields to decrease more than positive slopes of regression lines for small yields.

As described in the *Introduction*, breeding for robustness for both milk production and health traits is an issue in dairy cattle. In this study only milk production traits were considered. On the basis of our results, it seems that simultaneously breeding for an increase in milk GP and a decrease in ES by applying marker-assisted selection is difficult because no SNPs showed opposite directions of the effects. Genomic selection can be seen as marker assisted selection on a genome-wide scale. It is currently implemented in many dairy cattle populations (Goddard and Hayes 2009). Improving ES by genomic selection should be possible by considering ES as an additional trait and by estimating genomic assisted breeding values for this trait. A reference population for the estimation of marker effects is needed. Existing reference populations mainly built by progeny-tested bulls can also be used for ES, provided that the daughters are distributed over a wide range of environments. As done in this study, the daughter records can then be used for the estimation of sire effects for ES, which in turn, can be used to estimate marker effects. The most appropriate method for this estimation depends on the genetic architecture of the trait, *i.e.*, on the number of genes affecting the trait and on the distribution of the effect size (Hayes *et al.* 2010). The current study shows that for the estimation of marker effects for ES a model should be used that is tailored to traits affected by many genes with small effects and few with large effects.

We presented GxE for milk traits resulting in substantial scaling effects. Many SNP clusters affecting GP and ES could be identified and validated. The effects of some SNPs for ES were not removable by a data transformation, indicating that these are not solely scaling effects. The positions of these clusters were often found in well-known candidate regions affecting milk traits. No validated SNP showed effects for ES and GP in opposite directions. We showed that ES of milk traits is a typical quantitative trait controlled by many genes with small and few genes with large effects.

## Supplementary Material

Supporting Information
