# Supplementary material for: Using Genome-Wide Association Analysis to Characterize Environmental Sensitivity of Milk Traits in Dairy Cattle
Source: G3 (Bethesda). 2013 Jul 1;3(7):1085–93. doi: 10.1534/g3.113.006536 (PMC3704237; doi:10.1534/g3.113.006536)
Supplement: Supporting Information [file supp_3_7_1085__index.html]

Using Genome-Wide Association Analysis to Characterize Environmental Sensitivity of Milk Traits in Dairy Cattle — Supporting Information 

# Using Genome-Wide Association Analysis to Characterize Environmental Sensitivity of Milk Traits in Dairy Cattle

## Supporting Information for Streit *et al.*, 2013

**Files in this Data Supplement:**

- Supporting Information - Figure S1, Files S1-S4, and Tables S1-S2 (PDF, 1 MB)
- Figure S1 - Histogram of the environmental descriptor milk energy yield (PDF, 200 KB)
- Table S1 - Validated SNPs with chromosome (BTA), position in base pairs (bp), F-values and effects for intercept and slope (PDF, 489 KB)
- Table S2 - Validated SNPs with chromosome (BTA), position in base pairs (bp), F-values and effects for intercept and slope (PDF, 468 KB)
- File S1 - Map data (.zip, 406 KB)
- File S2 - Genotype data (.zip, 23 MB)
- File S3 - Pedigree data (.zip, 33 KB)
- File S4 - Phenotype data (.zip, 215 MB)
