# Supplementary figures and images for: Using Genome-Wide Association Analysis to Characterize Environmental Sensitivity of Milk Traits in Dairy Cattle
Source: G3 (Bethesda). 2013 Jul 1;3(7):1085–93. doi: 10.1534/g3.113.006536 (PMC3704237; doi:10.1534/g3.113.006536)

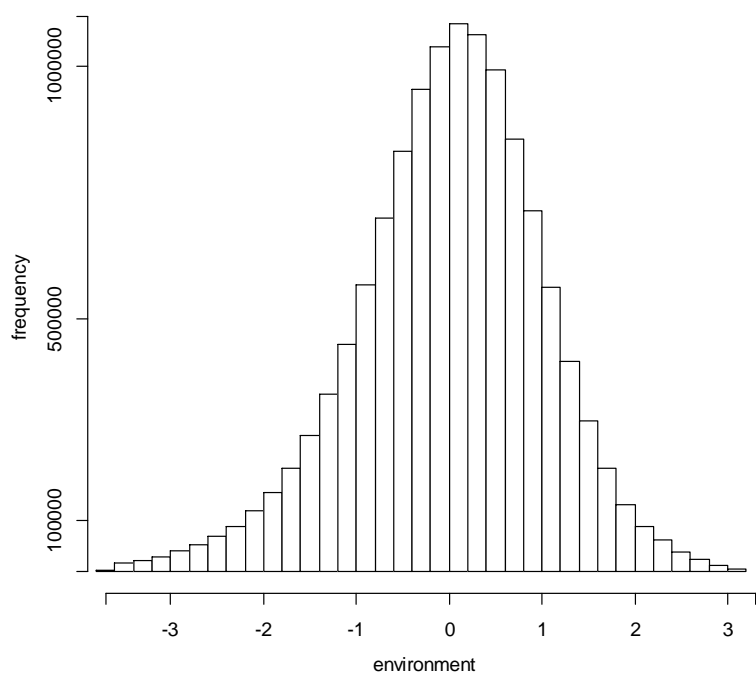

**Figure S1** Histogram of the environmental descriptor milk energy yield.

Supplement: Supporting Information [file supp_g3.113.006536_FigureS1.pdf]
