# Supplementary material for: Using Genome-Wide Association Analysis to Characterize Environmental Sensitivity of Milk Traits in Dairy Cattle
Source: G3 (Bethesda). 2013 Jul 1;3(7):1085–93. doi: 10.1534/g3.113.006536 (PMC3704237; doi:10.1534/g3.113.006536)
Supplement: Supporting Information [file supp_g3.113.006536_TableS1.pdf]

Table S1 Validated SNPs with chromosome (BTA), position in base pairs (bp), F-values and effects for intercept and slope. Effect estimates were taken from the validation set. Validated SNPs are indicated in bold type F-values and effect estimates.

			F-values in discovery dataset		Effects (in σ)	
SNP name	BTA	bp	Intercept	Slope	Intercept	Slope
**Protein yield**						
ARS-BFGL-NGS-54077	1	87338372	7.09	**10.96**	0.094	**0.148**
ARS-BFGL-BAC-7205	1	120983738	**15.75**	**16.35**	**0.117**	**0.104**
ARS-BFGL-NGS-99492	1	121607486	**16.95**	**16.54**	**0.125**	**0.126**
ARS-BFGL-BAC-13578	1	121811393	**13.78**	7.48	**0.121**	0.102
ARS-BFGL-NGS-98257	1	127680108	**13.65**	4.44	**0.109**	0.075
ARS-BFGL-NGS-86079	2	19126180	**15.75**	6.79	**0.136**	0.114
Hapmap53232-rs29020795	2	19202356	**14.56**	9.90	**0.130**	0.109
Hapmap43615-BTA-54400	2	19255900	**11.87**	7.53	**0.139**	0.106
Hapmap60669-rs29018484	2	20687353	**16.88**	15.06	**0.222**	0.140
BTA-101354-no-rs	2	58818593	10.96	**11.72**	0.067	**0.116**
Hapmap28102-BTA-152636	2	58842740	10.84	**11.37**	0.069	**0.116**
ARS-BFGL-NGS-113152	4	14644971	**10.88**	8.87	**0.143**	0.139
ARS-BFGL-NGS-68464	5	18395406	**12.71**	10.07	**0.145**	0.090
Hapmap33079-BTA-163567	6	1936	**15.61**	5.94	**0.142**	0.068
Hapmap54442-rs29025673	6	31579806	**14.14**	8.19	**0.102**	0.092
Hapmap27701-BTC-050761	6	40183166	**12.68**	8.34	**0.126**	0.094
Hapmap31819-BTA-156590	6	40421547	**13.80**	9.11	**0.126**	0.094
Hapmap23201-BTC-072836	6	40655229	**21.27**	17.26	**0.113**	0.095
Hapmap32946-BTC-046820	6	41129701	**27.71**	15.88	**0.137**	0.086
ARS-BFGL-NGS-39570	6	46320087	**14.72**	3.26	**0.169**	0.159
ARS-BFGL-NGS-43679	6	109680595	**13.67**	4.00	**0.140**	0.054
BTB-00281303	6	111612203	**15.07**	2.41	**0.153**	0.090
ARS-BFGL-NGS-113181	7	62800839	**12.32**	10.78	**0.129**	0.108
ARS-BFGL-NGS-113819	7	63609102	**15.25**	6.29	**0.153**	0.089
ARS-BFGL-NGS-109819	7	63664393	**15.72**	5.91	**0.154**	0.086
ARS-BFGL-NGS-4062	8	5505897	6.17	**11.71**	0.116	**0.122**
Hapmap44053-BTA-28733	8	6369477	**15.90**	**14.16**	**0.118**	**0.133**
BTB-00389006	9	39062436	16.19	**16.89**	0.097	**0.124**
BTA-06997-rs29021351	9	40153426	**24.71**	22.35	**0.220**	0.154
BTA-83317-no-rs	9	40393986	**26.22**	21.89	**0.114**	0.081
BTA-83528-no-rs	9	41691114	**25.23**	18.48	**0.170**	0.112
ARS-BFGL-NGS-37982	9	44200288	**22.50**	15.00	**0.105**	0.084
ARS-BFGL-NGS-52530	9	44230587	**25.27**	**25.92**	**0.215**	**0.185**
ARS-BFGL-NGS-103934	9	44255942	**17.63**	17.33	**0.162**	0.115
Hapmap57331-rs29009884	9	45457232	14.35	**12.31**	0.072	**0.096**
ARS-BFGL-NGS-75844	9	45497534	14.31	**12.54**	0.072	**0.096**
ARS-BFGL-NGS-39444	9	45590254	14.31	**12.54**	0.072	**0.096**
BTA-10828-no-rs	9	46600974	24.59	**23.15**	0.096	**0.105**
BTA-83605-no-rs	9	48362593	**13.56**	10.43	**0.116**	0.103
BTB-00391835	9	52160813	**11.20**	5.70	**0.157**	0.085
ARS-BFGL-NGS-25071	9	75512134	7.97	**11.90**	0.051	**0.107**
ARS-BFGL-NGS-80176	10	64041482	**12.30**	9.49	**0.121**	0.129
ARS-BFGL-NGS-88689	11	29901111	7.53	**12.41**	0.123	**0.143**
ARS-BFGL-NGS-87426	11	30070765	**11.73**	**12.74**	**0.114**	**0.127**
ARS-BFGL-NGS-21332	11	30108643	8.61	**12.87**	0.120	**0.132**
ARS-BFGL-NGS-118724	11	30366110	**12.45**	10.44	**0.107**	0.121
ARS-BFGL-NGS-112015	13	63235775	**13.16**	2.09	**0.108**	0.050
Hapmap54034-rs29026486	13	65183781	**11.97**	4.07	**0.106**	0.073

ARS-BFGL-NGS-63777	13	67075815	**21.53**	**17.56**	**0.200**	**0.157**
ARS-BFGL-NGS-103635	13	67816926	**11.00**	1.02	**0.104**	0.059
ARS-BFGL-NGS-52851	13	77352863	11.87	**16.39**	0.048	**0.110**
Hapmap38308-BTA-33903	13	77756882	4.13	**11.00**	0.045	**0.102**
Hapmap30383-BTC-005848	14	76704	**45.65**	**31.66**	**0.168**	**0.134**
BTA-34956-no-rs	14	101474	**20.91**	7.85	**0.129**	0.112
ARS-BFGL-NGS-57820	14	236533	**72.24**	**56.71**	**0.193**	**0.148**
ARS-BFGL-NGS-34135	14	260342	**33.61**	18.35	**0.114**	0.095
ARS-BFGL-NGS-94706	14	281534	**32.30**	17.16	**0.118**	0.093
ARS-BFGL-NGS-4939	14	443936	**76.22**	**55.93**	**0.189**	**0.138**
ARS-BFGL-NGS-107379	14	679601	**61.01**	**51.65**	**0.158**	**0.122**
Hapmap25384-BTC-001997	14	835055	**27.13**	16.99	**0.117**	0.070
Hapmap24715-BTC-001973	14	856890	**22.88**	13.75	**0.116**	0.073
ARS-BFGL-NGS-103064	14	1193335	**23.15**	9.03	**0.109**	0.060
Hapmap25486-BTC-072553	14	1285036	**19.13**	8.43	**0.115**	0.083
ARS-BFGL-BAC-20965	14	5225005	**14.28**	8.91	**0.136**	0.104
Hapmap23851-BTC-048718	14	5387835	**17.21**	16.92	**0.126**	0.095
BTB-01988444	14	56535371	3.95	**11.67**	0.092	**0.126**
ARS-BFGL-NGS-100131	15	21041772	9.75	**11.06**	0.099	**0.132**
BTB-01698088	16	9738423	11.05	**16.13**	0.086	**0.139**
ARS-BFGL-NGS-56645	16	23920210	**15.37**	5.10	**0.117**	0.092
BTA-38367-no-rs	16	26379579	**12.89**	9.03	**0.096**	0.082
ARS-BFGL-NGS-41039	16	27619045	**14.91**	5.09	**0.183**	0.109
ARS-BFGL-NGS-38023	16	33318455	**18.15**	9.91	**0.107**	0.104
ARS-BFGL-NGS-26559	16	33367687	**11.43**	6.70	**0.124**	0.112
ARS-BFGL-NGS-59645	16	73117625	**15.94**	10.48	**0.124**	0.092
BTA-97501-no-rs	18	57095120	**16.33**	8.05	**0.108**	0.069
ARS-BFGL-NGS-15837	18	62533851	**11.11**	4.42	**0.110**	0.118
ARS-BFGL-BAC-33672	20	44108671	10.68	**12.05**	0.152	**0.185**
BTA-17135-no-rs	20	49557519	11.88	**16.26**	0.132	**0.175**
BTA-41516-no-rs	20	49593049	10.63	**15.64**	0.117	**0.165**
BTB-01251603	20	50418473	9.19	**12.88**	0.172	**0.198**
ARS-BFGL-BAC-34291	20	54837742	8.66	**13.62**	0.123	**0.152**
Hapmap53927-rs29025287	20	56464296	8.94	**13.45**	0.135	**0.179**
BTB-00787949	20	56494412	5.89	**12.27**	0.150	**0.186**
ARS-BFGL-BAC-36842	20	58820614	6.51	**14.46**	0.097	**0.146**
ARS-BFGL-NGS-38258	20	60185080	**11.42**	7.68	**0.138**	0.119
BTA-12959-no-rs	21	10922512	**13.46**	9.60	**0.114**	0.104
ARS-BFGL-NGS-101900	21	30314497	**14.12**	4.85	**0.106**	0.099
ARS-BFGL-NGS-110044	21	30892171	**11.81**	2.78	**0.158**	0.143
ARS-BFGL-NGS-55374	25	28795160	**24.40**	**25.01**	**0.215**	**0.185**
ARS-BFGL-NGS-2464	26	18709176	**20.40**	10.13	**0.098**	0.097
ARS-BFGL-NGS-71584	26	18863914	28.46	**15.95**	0.091	**0.102**
ARS-BFGL-NGS-113339	26	19918015	**16.35**	7.34	**0.105**	0.083
BTA-62184-no-rs	26	20014035	**17.65**	9.58	**0.118**	0.099
BTA-60778-no-rs	26	20090833	**21.14**	**14.75**	**0.127**	**0.117**
BTB-00932332	26	22551770	15.48	**11.32**	0.101	**0.130**
ARS-BFGL-NGS-107403	26	23470277	18.11	**12.42**	0.098	**0.122**
Hapmap28763-BTA-162328	26	26472420	12.31	**12.37**	0.085	**0.109**
BTB-01622498	28	1436040	3.42	**12.49**	0.100	**0.158**
ARS-BFGL-NGS-119076	28	15361777	**12.02**	8.12	**0.126**	0.086
ARS-BFGL-NGS-43501	28	17344828	14.42	**17.58**	0.083	**0.136**
ARS-BFGL-NGS-118693	28	17493199	10.68	**13.14**	0.139	**0.127**
**Fat yield**						
Hapmap38956-BTA-43309	1	98853038	**12.91**	10.82	**0.101**	0.114

BTB-01562245	1	113644592	**11.25**	**11.71**	**0.149**	**0.175**
BTA-104132-no-rs	1	113669783	**11.95**	**12.23**	**0.151**	**0.178**
ARS-BFGL-BAC-7205	1	120983738	**12.64**	12.33	**0.079**	0.072
ARS-BFGL-NGS-88388	2	13718482	**19.97**	**18.86**	**0.076**	**0.081**
ARS-BFGL-NGS-112315	2	40959609	**12.86**	5.12	**0.093**	0.091
ARS-BFGL-NGS-44416	2	48557519	**11.31**	9.45	**0.089**	0.082
ARS-BFGL-NGS-8503	2	87880854	**11.07**	**11.84**	**0.117**	**0.095**
BTB-00108243	2	112019423	**17.65**	**21.62**	**0.137**	**0.164**
BTB-01678000	3	6985014	**20.43**	20.37	**0.077**	0.068
BTB-01678060	3	7009487	**20.08**	**21.54**	**0.092**	**0.091**
ARS-BFGL-NGS-5956	3	8558755	**18.73**	**14.50**	**0.092**	**0.093**
ARS-BFGL-NGS-112616	3	8598511	**17.12**	**12.48**	**0.085**	**0.085**
ARS-BFGL-NGS-102139	3	24018818	9.79	**11.56**	0.067	**0.078**
Hapmap50814-BTA-89905	3	41486200	**11.97**	9.50	**0.095**	0.079
BTB-01851577	3	50093239	**11.69**	6.77	**0.089**	0.072
INRA-648	3	54347354	13.39	**11.31**	0.147	**0.168**
Hapmap43441-BTA-103289	3	61621627	17.59	**20.74**	0.068	**0.083**
BTA-68164-no-rs	3	68215262	**13.11**	8.02	**0.095**	0.062
BTB-00131847	3	68241075	**13.16**	8.09	**0.097**	0.066
BTA-54952-no-rs	4	11830564	**11.96**	9.08	**0.089**	0.090
ARS-BFGL-NGS-20815	4	15021946	**15.91**	14.68	**0.083**	0.076
ARS-BFGL-NGS-117196	4	53206872	9.84	**12.44**	0.073	**0.085**
ARS-BFGL-NGS-110647	4	77565085	9.95	**12.41**	0.081	**0.093**
Hapmap39895-BTA-15668	5	15392995	**11.42**	7.32	**0.093**	0.081
ARS-BFGL-NGS-108617	5	98082173	**15.12**	**11.57**	**0.107**	**0.110**
ARS-BFGL-NGS-95906	5	100351926	**20.33**	**17.38**	**0.090**	**0.091**
Hapmap53294-rs29016908	5	101090418	**40.43**	**22.97**	**0.086**	**0.095**
Hapmap60021-ss46526426	5	101979581	**21.19**	**13.64**	**0.095**	**0.088**
BTB-00270281	6	95770023	**16.38**	12.12	**0.095**	0.073
Hapmap58150-rs29020620	6	96724594	**11.78**	8.53	**0.076**	0.070
BTB-01700063	6	99086447	**21.66**	**16.75**	**0.114**	**0.102**
Hapmap53916-rs29021982	6	99581269	**18.46**	**15.67**	**0.094**	**0.094**
Hapmap48078-BTA-77495	6	99827767	**13.22**	**14.54**	**0.076**	**0.079**
ARS-BFGL-NGS-14880	7	53879989	**14.95**	**17.58**	**0.115**	**0.104**
ARS-BFGL-NGS-113181	7	62800839	**17.55**	**20.39**	**0.077**	**0.080**
BTB-02035459	7	63196194	**11.52**	8.67	**0.073**	0.073
BTB-01219396	7	63221359	**11.52**	8.67	**0.073**	0.073
ARS-BFGL-NGS-113819	7	63609102	12.18	**13.22**	0.071	**0.081**
ARS-BFGL-NGS-109819	7	63664393	12.02	**12.76**	0.073	**0.083**
BTA-12616-no-rs	7	64712171	**12.81**	8.91	**0.074**	0.063
ARS-BFGL-NGS-65419	7	66102696	**15.93**	**13.18**	**0.092**	**0.094**
ARS-BFGL-NGS-12863	7	68960712	**16.23**	7.43	**0.105**	0.092
ARS-BFGL-NGS-23091	7	69342623	**13.06**	6.26	**0.110**	0.095
BTB-01222854	7	74587278	**13.21**	**11.20**	**0.105**	**0.104**
BTB-01321253	7	83625073	**19.55**	**20.56**	**0.083**	**0.077**
Hapmap44053-BTA-28733	8	6369477	**15.07**	**12.22**	**0.096**	**0.113**
BTA-102639-no-rs	8	29764842	7.33	**11.04**	0.083	**0.105**
ARS-BFGL-NGS-68597	8	32188298	9.59	**12.15**	0.144	**0.139**
BTB-01184997	8	36171902	**11.48**	10.62	**0.097**	0.092
Hapmap31805-BTA-154485	8	36232703	**12.95**	**13.66**	**0.078**	**0.084**
Hapmap41758-BTA-116042	8	37197678	**11.23**	7.67	**0.105**	0.105
BTB-00384442	9	22407445	**11.25**	8.57	**0.094**	0.079
BTA-83317-no-rs	9	40393986	**18.16**	16.64	**0.087**	0.077
BTA-83528-no-rs	9	41691114	**17.66**	15.90	**0.132**	0.115
Hapmap34441-BES9_Contig154_536	9	73154820	**11.99**	10.47	**0.084**	0.091
UA-IFASA-2589	9	82175488	9.65	**11.75**	0.072	**0.086**

Hapmap30370-BTA-99862	11	14499391	**14.20**	8.56	**0.104**	0.100
Hapmap29758-BTC-003619	14	5261	**39.73**	**33.19**	**0.112**	**0.109**
Hapmap30381-BTC-005750	14	50873	**59.46**	**54.84**	**0.129**	**0.129**
Hapmap30383-BTC-005848	14	76704	**65.31**	**55.73**	**0.132**	**0.132**
BTA-34956-no-rs	14	101474	**29.89**	**29.89**	**0.106**	**0.104**
ARS-BFGL-NGS-57820	14	236533	**147.55**	**121.82**	**0.194**	**0.195**
ARS-BFGL-NGS-34135	14	260342	**79.17**	**71.47**	**0.146**	**0.144**
ARS-BFGL-NGS-94706	14	281534	**75.70**	**68.83**	**0.143**	**0.141**
ARS-BFGL-NGS-4939	14	443936	**159.02**	**132.41**	**0.206**	**0.208**
ARS-BFGL-NGS-71749	14	596340	**41.77**	**36.70**	**0.144**	**0.147**
ARS-BFGL-NGS-107379	14	679601	**101.31**	**78.31**	**0.163**	**0.160**
ARS-BFGL-NGS-18365	14	741868	**46.48**	**33.09**	**0.084**	**0.094**
Hapmap30922-BTC-002021	14	763332	**49.05**	**35.93**	**0.087**	**0.101**
Hapmap25384-BTC-001997	14	835055	**63.54**	**55.55**	**0.103**	**0.119**
Hapmap24715-BTC-001973	14	856890	**60.80**	**54.16**	**0.105**	**0.120**
BTA-35941-no-rs	14	894253	**97.39**	**78.45**	**0.155**	**0.156**
ARS-BFGL-NGS-101653	14	931163	**41.64**	**36.14**	**0.133**	**0.144**
ARS-BFGL-NGS-26520	14	996983	**69.35**	**59.88**	**0.149**	**0.159**
UA-IFASA-6878	14	1044040	**36.43**	26.64	**0.074**	0.071
ARS-BFGL-NGS-22866	14	1131951	**44.94**	**42.65**	**0.126**	**0.119**
ARS-BFGL-NGS-103064	14	1193335	**61.80**	**58.85**	**0.110**	**0.138**
ARS-BFGL-NGS-3122	14	1264232	**33.56**	**31.89**	**0.092**	**0.126**
Hapmap25486-BTC-072553	14	1285036	**34.57**	**35.23**	**0.108**	**0.121**
Hapmap30646-BTC-002054	14	1461084	**68.94**	**51.83**	**0.142**	**0.142**
Hapmap30086-BTC-002066	14	1490177	**110.60**	**85.69**	**0.147**	**0.152**
Hapmap30374-BTC-002159	14	1546590	**89.52**	**73.07**	**0.153**	**0.154**
ARS-BFGL-NGS-74378	14	1889209	**48.29**	**35.14**	**0.099**	**0.107**
ARS-BFGL-NGS-117542	14	1913107	**28.30**	**24.11**	**0.103**	**0.105**
UA-IFASA-9288	14	2201869	**48.59**	**32.70**	**0.089**	**0.103**
Hapmap24777-BTC-064977	14	2261622	**14.06**	**12.67**	**0.079**	**0.095**
Hapmap32970-BTC-064990	14	2288509	**31.99**	**24.55**	**0.080**	**0.095**
Hapmap24986-BTC-065021	14	2313594	**31.99**	**24.55**	**0.080**	**0.095**
ARS-BFGL-NGS-22111	14	2347218	19.48	**18.07**	0.059	**0.079**
UA-IFASA-7269	14	2370255	19.48	**18.07**	0.059	**0.079**
Hapmap26072-BTC-065132	14	2391825	24.28	**24.38**	0.070	**0.087**
ARS-BFGL-NGS-113575	14	2484498	**35.10**	**30.78**	**0.085**	**0.097**
ARS-BFGL-NGS-118081	14	2511264	**41.29**	**37.93**	**0.113**	**0.116**
ARS-BFGL-NGS-56327	14	2580413	**60.32**	**50.89**	**0.105**	**0.116**
ARS-BFGL-NGS-100480	14	2607582	**75.52**	**63.36**	**0.121**	**0.128**
UA-IFASA-5306	14	2711614	**48.24**	**32.60**	**0.099**	**0.110**
Hapmap27703-BTC-053907	14	2826072	27.55	**23.02**	0.063	**0.081**
Hapmap22692-BTC-068210	14	3018725	**36.91**	**23.96**	**0.080**	**0.086**
Hapmap23302-BTC-052123	14	3099634	**36.05**	**20.28**	**0.090**	**0.096**
UA-IFASA-6329	14	3465238	**25.41**	**16.40**	**0.094**	**0.083**
ARS-BFGL-NGS-3571	14	3587017	**26.21**	**18.53**	**0.099**	**0.101**
ARS-BFGL-NGS-110563	14	3799229	**25.68**	**15.99**	**0.103**	**0.105**
Hapmap32262-BTC-066621	14	3834070	**13.39**	8.84	**0.087**	0.083
ARS-BFGL-NGS-115947	14	3865963	**34.05**	**19.86**	**0.107**	**0.109**
Hapmap30988-BTC-056315	14	4693900	**20.44**	**15.88**	**0.097**	**0.091**
ARS-BFGL-NGS-110894	14	5282437	**15.13**	7.87	**0.094**	0.101
UA-IFASA-6647	14	5808643	**21.59**	12.18	**0.072**	0.073
ARS-BFGL-NGS-102953	14	5867265	**19.06**	9.11	**0.076**	0.078
ARS-BFGL-NGS-37911	14	14274020	**11.29**	9.61	**0.090**	0.096
ARS-BFGL-NGS-16622	15	64781292	**11.71**	9.29	**0.089**	0.085
BTA-37324-no-rs	15	64802082	**10.94**	8.43	**0.091**	0.083
Hapmap54310-rs29012181	16	8166691	9.72	**12.29**	0.093	**0.092**

Hapmap43402-BTA-91283	16	8249735	8.15	**11.65**	0.086	**0.088**
ARS-BFGL-NGS-56645	16	23920210	**11.80**	8.21	**0.082**	0.073
ARS-BFGL-NGS-26559	16	33367687	**11.77**	10.10	**0.089**	0.094
ARS-BFGL-NGS-93660	16	77541689	**12.02**	12.76	**0.088**	0.073
ARS-BFGL-NGS-18128	17	22770146	**11.24**	8.71	**0.096**	0.100
ARS-BFGL-NGS-91287	18	10052638	**17.13**	**20.25**	**0.117**	**0.101**
ARS-BFGL-NGS-111247	19	43146804	**14.32**	15.09	**0.075**	0.072
ARS-BFGL-NGS-24479	19	45901285	**11.09**	9.17	**0.092**	0.087
ARS-BFGL-NGS-113693	19	45926259	**12.27**	10.29	**0.092**	0.087
BTB-00783355	20	43550938	10.23	**16.06**	0.087	**0.111**
Hapmap48608-BTA-111028	20	52535573	12.29	**16.19**	0.076	**0.088**
BTA-12959-no-rs	21	10922512	**12.61**	11.88	**0.074**	0.071
ARS-BFGL-NGS-101900	21	30314497	**12.99**	6.17	**0.073**	0.066
ARS-BFGL-NGS-39397	26	21166268	**12.98**	10.82	**0.078**	0.081
Hapmap46411-BTA-15820	26	21404446	**14.10**	**12.01**	**0.094**	**0.103**
Hapmap31825-BTA-158647	26	21476707	**15.45**	**11.71**	**0.073**	**0.079**
ARS-BFGL-NGS-110077	26	21729361	**13.86**	**14.13**	**0.086**	**0.086**
ARS-BFGL-NGS-18603	26	21853286	21.66	**22.77**	0.067	**0.078**
ARS-BFGL-NGS-116481	26	22411701	**22.81**	**22.25**	**0.077**	**0.079**
Hapmap24832-BTA-138805	26	22449570	**23.72**	**23.06**	**0.077**	**0.079**
ARS-BFGL-NGS-6259	26	22492302	**21.27**	**20.72**	**0.077**	**0.079**
BTB-00932332	26	22551770	22.91	**21.24**	0.073	**0.084**
ARS-BFGL-NGS-1092	26	24837303	**17.02**	**19.39**	**0.096**	**0.093**
UA-IFASA-4715	26	25330026	16.24	**20.09**	0.063	**0.081**
ARS-BFGL-NGS-38386	26	32836475	7.14	**11.37**	0.065	**0.103**
ARS-BFGL-NGS-105944	26	34196569	**11.04**	**11.12**	**0.106**	**0.109**
ARS-BFGL-NGS-53731	26	37801879	13.09	**15.19**	0.062	**0.087**
ARS-USMARC-Parent-EF034086-no-rs	26	38309860	16.12	**17.87**	0.054	**0.075**
Hapmap35000-BES9_Contig272_944	26	38309861	16.12	**17.87**	0.054	**0.075**
ARS-BFGL-NGS-60822	28	17252660	16.04	**12.16**	0.066	**0.079**
UA-IFASA-6208	28	27379038	**12.33**	8.31	**0.081**	0.081
**Milk yield**						
ARS-BFGL-NGS-23720	1	38486179	4.91	**10.88**	0.146	**0.130**
ARS-BFGL-BAC-13578	1	121811393	**13.51**	**11.19**	**0.189**	**0.179**
ARS-BFGL-NGS-86079	2	19126180	**14.12**	9.49	**0.194**	0.182
Hapmap53232-rs29020795	2	19202356	**12.91**	**11.87**	**0.170**	**0.161**
Hapmap60669-rs29018484	2	20687353	**13.93**	12.24	**0.324**	0.249
ARS-BFGL-NGS-23786	2	22727721	**12.86**	8.56	**0.170**	0.132
UA-IFASA-4555	2	103573252	**10.91**	7.81	**0.347**	0.267
ARS-BFGL-NGS-119065	3	56970477	8.93	**12.42**	0.280	**0.256**
ARS-BFGL-NGS-75548	4	78246158	**15.43**	**11.18**	**0.446**	**0.338**
BTA-74090-no-rs	5	7569039	9.55	**12.21**	0.178	**0.147**
Hapmap33079-BTA-163567	6	1936	**13.03**	9.90	**0.175**	0.098
ARS-BFGL-NGS-117147	6	31235325	**10.90**	3.52	**0.199**	0.135
BTA-75680-no-rs	6	31470687	**11.66**	6.98	**0.157**	0.102
Hapmap54442-rs29025673	6	31579806	**12.51**	8.27	**0.157**	0.102
Hapmap31819-BTA-156590	6	40421547	10.20	**11.60**	0.165	**0.124**
UA-IFASA-2111	6	85289127	**14.59**	15.14	**0.192**	0.122
Hapmap25708-BTC-043671	6	88263655	**11.16**	11.06	**0.213**	0.111
Hapmap40845-BTA-97263	6	92788189	**11.94**	7.87	**0.196**	0.143
BTB-01428914	6	93850918	**12.79**	8.07	**0.157**	0.098
BTB-00281303	6	111612203	**10.87**	3.17	**0.209**	0.093
Hapmap53417-rs29014877	7	106039868	**13.08**	11.74	**0.164**	0.114
ARS-BFGL-NGS-4062	8	5505897	6.84	**11.25**	0.199	**0.183**
Hapmap44053-BTA-28733	8	6369477	13.69	**11.43**	0.151	**0.166**

BTA-82314-no-rs	8	97201103	9.03	**15.81**	0.162	**0.172**
ARS-BFGL-NGS-52530	9	44230587	**12.72**	10.89	**0.273**	0.192
Hapmap49359-BTA-88301	9	45435722	8.60	**11.76**	0.147	**0.149**
ARS-BFGL-NGS-75844	9	45497534	7.87	**11.04**	0.133	**0.150**
ARS-BFGL-NGS-39444	9	45590254	7.87	**11.04**	0.133	**0.150**
BTA-10828-no-rs	9	46600974	**15.86**	**18.25**	**0.160**	**0.150**
ARS-BFGL-NGS-25071	9	75512134	9.95	**13.07**	0.083	**0.137**
ARS-BFGL-NGS-62628	9	82136926	10.06	**11.58**	0.149	**0.133**
ARS-BFGL-NGS-105675	11	104891662	11.47	**14.14**	0.145	**0.156**
ARS-BFGL-NGS-78549	11	106534689	**12.38**	**14.06**	**0.234**	**0.220**
ARS-BFGL-NGS-56157	13	63208626	**13.95**	5.96	**0.163**	0.088
Hapmap54034-rs29026486	13	65183781	**14.87**	4.39	**0.188**	0.090
ARS-BFGL-NGS-103635	13	67816926	**13.47**	4.35	**0.157**	0.081
Hapmap44949-BTA-33430	13	67920496	**11.12**	5.80	**0.209**	0.119
Hapmap29758-BTC-003619	14	5261	**41.37**	**45.38**	**0.175**	**0.156**
Hapmap30381-BTC-005750	14	50873	**46.72**	**39.87**	**0.280**	**0.254**
Hapmap30383-BTC-005848	14	76704	**130.39**	**113.46**	**0.373**	**0.292**
BTA-34956-no-rs	14	101474	**69.39**	**51.08**	**0.287**	**0.246**
ARS-BFGL-NGS-57820	14	236533	**218.33**	**207.08**	**0.478**	**0.372**
ARS-BFGL-NGS-34135	14	260342	**111.86**	**96.34**	**0.305**	**0.261**
ARS-BFGL-NGS-94706	14	281534	**109.45**	**91.11**	**0.311**	**0.259**
ARS-BFGL-NGS-4939	14	443936	**226.90**	**210.30**	**0.477**	**0.371**
ARS-BFGL-NGS-71749	14	596340	**32.17**	24.98	**0.205**	0.153
ARS-BFGL-NGS-107379	14	679601	**165.98**	**159.61**	**0.387**	**0.310**
ARS-BFGL-NGS-18365	14	741868	**36.88**	44.68	**0.202**	0.115
Hapmap30922-BTC-002021	14	763332	**27.70**	33.83	**0.187**	0.111
Hapmap25384-BTC-001997	14	835055	**86.29**	**71.08**	**0.294**	**0.201**
Hapmap24715-BTC-001973	14	856890	**76.90**	**63.03**	**0.290**	**0.203**
BTA-35941-no-rs	14	894253	**81.51**	**91.67**	**0.245**	**0.196**
ARS-BFGL-NGS-101653	14	931163	**48.62**	**43.61**	**0.228**	**0.173**
ARS-BFGL-NGS-26520	14	996983	**55.92**	**51.37**	**0.211**	**0.151**
UA-IFASA-6878	14	1044040	**82.94**	**84.88**	**0.254**	**0.200**
ARS-BFGL-NGS-22866	14	1131951	**53.32**	**49.99**	**0.190**	**0.190**
ARS-BFGL-NGS-103064	14	1193335	**78.12**	**56.27**	**0.288**	**0.196**
ARS-BFGL-NGS-3122	14	1264232	**56.10**	**39.10**	**0.262**	**0.155**
Hapmap25486-BTC-072553	14	1285036	**54.16**	**34.14**	**0.269**	**0.185**
Hapmap30646-BTC-002054	14	1461084	**61.98**	**70.96**	**0.216**	**0.177**
Hapmap30086-BTC-002066	14	1490177	**77.59**	**89.06**	**0.244**	**0.198**
Hapmap30374-BTC-002159	14	1546590	**84.37**	**91.65**	**0.236**	**0.193**
ARS-BFGL-NGS-74378	14	1889209	**51.60**	**62.95**	**0.264**	**0.209**
ARS-BFGL-NGS-117542	14	1913107	**37.52**	**38.66**	**0.239**	**0.201**
UA-IFASA-9288	14	2201869	**34.84**	**42.80**	**0.209**	**0.147**
Hapmap32970-BTC-064990	14	2288509	**19.04**	19.18	**0.165**	0.102
Hapmap24986-BTC-065021	14	2313594	**19.04**	19.18	**0.165**	0.102
Hapmap26072-BTC-065132	14	2391825	**37.91**	**27.05**	**0.221**	**0.173**
Hapmap26527-BTC-005059	14	2418618	**26.38**	**22.86**	**0.175**	**0.140**
ARS-BFGL-NGS-113575	14	2484498	**15.20**	**18.65**	**0.173**	**0.136**
ARS-BFGL-NGS-118081	14	2511264	**27.04**	**26.10**	**0.189**	**0.167**
ARS-BFGL-NGS-56327	14	2580413	**37.86**	**38.51**	**0.224**	**0.179**
ARS-BFGL-NGS-100480	14	2607582	**47.61**	**45.61**	**0.243**	**0.192**
ARS-BFGL-NGS-42263	14	2681399	**23.44**	**18.82**	**0.197**	**0.165**
UA-IFASA-5306	14	2711614	**36.83**	**45.80**	**0.223**	**0.171**
ARS-BFGL-NGS-54400	14	2736946	**21.12**	**16.78**	**0.206**	**0.163**
Hapmap22783-BTC-068255	14	2989274	**25.42**	**32.59**	**0.207**	**0.178**
Hapmap22692-BTC-068210	14	3018725	**34.59**	**45.38**	**0.199**	**0.156**
Hapmap23302-BTC-052123	14	3099634	**36.42**	**52.77**	**0.206**	**0.161**

Hapmap25217-BTC-067767	14	3189311	**35.96**	24.53	**0.189**	0.094
UA-IFASA-6329	14	3465238	**25.58**	**29.83**	**0.166**	**0.133**
ARS-BFGL-NGS-56339	14	3498808	**15.52**	**20.81**	**0.160**	**0.155**
UA-IFASA-8927	14	3640095	**18.48**	**18.50**	**0.154**	**0.130**
Hapmap32262-BTC-066621	14	3834070	**28.92**	**27.90**	**0.193**	**0.140**
Hapmap30091-BTC-005211	14	3940999	**26.82**	**23.11**	**0.225**	**0.149**
ARS-BFGL-BAC-24839	14	3993201	**22.12**	**26.61**	**0.164**	**0.137**
ARS-BFGL-BAC-24804	14	4157676	**42.02**	**59.28**	**0.163**	**0.136**
Hapmap51646-BTA-86764	14	4302230	**33.30**	41.88	**0.186**	0.111
ARS-BFGL-NGS-112858	14	4956374	**25.89**	**37.11**	**0.199**	**0.155**
Hapmap51078-BTA-87682	14	5064062	**15.80**	**12.92**	**0.192**	**0.127**
ARS-BFGL-NGS-55227	14	5085415	**19.63**	**33.44**	**0.163**	**0.145**
Hapmap32236-BTC-049785	14	5139497	19.37	**34.36**	0.142	**0.131**
ARS-BFGL-BAC-20965	14	5225005	**23.50**	**18.29**	**0.244**	**0.175**
Hapmap33635-BTC-049051	14	5318260	**16.46**	5.75	**0.192**	0.103
Hapmap27091-BTC-048823	14	5356987	**29.54**	27.13	**0.197**	0.113
Hapmap23851-BTC-048718	14	5387835	**28.66**	**24.81**	**0.236**	**0.131**
Hapmap32234-BTC-048199	14	5640337	**32.52**	**37.23**	**0.209**	**0.148**
Hapmap26283-BTC-048098	14	5696728	15.08	**30.24**	0.151	**0.128**
Hapmap32948-BTC-047992	14	5839289	**22.95**	**32.42**	**0.190**	**0.138**
Hapmap24756-BTC-047876	14	5915294	**20.39**	**19.47**	**0.215**	**0.158**
Hapmap25716-BTC-047850	14	5937549	**16.11**	**19.08**	**0.196**	**0.139**
Hapmap23799-BTC-047701	14	6044245	**11.39**	12.82	**0.212**	0.130
ARS-BFGL-BAC-8730	14	6252100	**30.95**	21.19	**0.187**	0.118
Hapmap53312-rs29018332	14	60576872	**20.09**	6.88	**0.159**	0.067
BTA-35525-no-rs	14	72400486	9.43	**11.65**	0.078	**0.154**
ARS-BFGL-BAC-23729	14	73127978	4.90	**11.73**	0.054	**0.168**
Hapmap43128-BTA-105550	15	52541506	**12.54**	7.43	**0.155**	0.084
Hapmap59019-rs29021918	18	41938135	10.02	**16.37**	0.118	**0.157**
ARS-BFGL-NGS-17557	20	1290829	**13.98**	11.50	**0.172**	0.127
Hapmap39811-BTA-122745	20	35432864	10.43	**13.05**	0.161	**0.165**
BTB-01888575	20	35457272	8.48	**11.86**	0.177	**0.185**
BTA-50244-no-rs	20	36466784	6.63	**12.12**	0.130	**0.167**
BTA-50402-no-rs	20	36667999	7.94	**15.52**	0.141	**0.179**
Hapmap26466-BTA-160199	20	36746234	8.57	**13.82**	0.151	**0.185**
BTA-50386-no-rs	20	36837402	6.13	**13.57**	0.134	**0.154**
BTA-50376-no-rs	20	36915967	**15.87**	**25.25**	**0.160**	**0.170**
Hapmap57531-rs29013890	20	36955574	**13.12**	**17.52**	**0.181**	**0.170**
Hapmap54884-rs29017180	20	50564369	8.80	**17.44**	0.174	**0.128**
ARS-BFGL-NGS-37182	22	5301596	**10.98**	10.56	**0.267**	0.273
ARS-BFGL-NGS-80066	23	20578210	**11.18**	7.83	**0.335**	0.200
Hapmap43294-BTA-56514	23	32759583	**12.29**	8.13	**0.303**	0.165
ARS-BFGL-NGS-55374	25	28795160	**12.07**	10.48	**0.273**	0.192
BTB-02094179	26	467952	7.37	**10.98**	0.183	**0.196**
ARS-BFGL-NGS-2127	26	13563198	**13.90**	8.58	**0.158**	0.115
ARS-BFGL-NGS-2464	26	18709176	**13.00**	8.73	**0.150**	0.134
ARS-BFGL-NGS-77668	26	18760372	19.50	**16.74**	0.132	**0.131**
ARS-BFGL-NGS-23064	26	18788121	18.84	**15.64**	0.132	**0.131**
ARS-BFGL-NGS-71584	26	18863914	19.45	**14.43**	0.113	**0.127**
BTB-00930720	26	21323659	**12.27**	**10.90**	**0.148**	**0.151**
Hapmap31825-BTA-158647	26	21476707	**14.00**	**11.18**	**0.160**	**0.135**
BTB-00931481	26	21631982	21.41	**17.08**	0.157	**0.157**
ARS-BFGL-NGS-18603	26	21853286	13.82	**14.96**	0.138	**0.139**
BTB-00932332	26	22551770	15.47	**14.23**	0.153	**0.177**
ARS-BFGL-NGS-107403	26	23470277	18.24	**16.31**	0.165	**0.181**
ARS-BFGL-NGS-119314	26	25634039	11.67	**14.68**	0.130	**0.148**

ARS-BFGL-NGS-109460	27	46280579	**14.33**	3.91	**0.172**	0.101
ARS-BFGL-NGS-116386	28	18996324	7.27	**15.71**	0.189	**0.148**
BTB-02080610	28	19938671	**12.70**	9.76	**0.201**	0.151
Hapmap58649-rs29011010	28	28185115	**15.70**	9.11	**0.154**	0.127
ARS-BFGL-NGS-116154	29	49020982	8.01	**10.91**	0.142	**0.165**
