# Supplementary material for: Using Genome-Wide Association Analysis to Characterize Environmental Sensitivity of Milk Traits in Dairy Cattle
Source: G3 (Bethesda). 2013 Jul 1;3(7):1085–93. doi: 10.1534/g3.113.006536 (PMC3704237; doi:10.1534/g3.113.006536)
Supplement: Supporting Information [file supp_g3.113.006536_TableS2.pdf]

Table S2 Validated SNPs with chromosome (BTA), position in base pairs (bp), F-values and effects for intercept and slope. Effect estimates were taken from the validation set. Validated SNPs are indicated in bold type F-values and effect estimates. Results from the analysis of the log-transformed data set.

			F-values in discovery dataset		Effects (in σ)	
SNP name	BTA	bp	Intercept	Slope	Intercept	Slope
**ln(Protein)**						
BTB-00014850	1	33047886	24.41	**22.24**	0.037	**0.120**
BTA-17929-no-rs	1	94762863	14.03	**10.87**	0.130	**0.157**
ARS-BFGL-BAC-7205	1	120983738	**15.50**	**13.42**	**0.190**	**0.124**
ARS-BFGL-NGS-99492	1	121607486	16.79	**14.24**	0.159	**0.144**
BTB-01116994	1	122957014	13.08	**12.97**	0.130	**0.171**
ARS-BFGL-NGS-5956	3	8558755	**11.21**	6.20	**0.225**	0.045
Hapmap22861-BTA-141421	3	60025569	10.41	**12.01**	0.093	**0.089**
Hapmap39895-BTA-15668	5	15392995	**14.67**	12.25	**0.219**	0.085
ARS-BFGL-NGS-68464	5	18395406	**12.53**	9.43	**0.229**	0.104
Hapmap23201-BTC-072836	6	40655229	21.27	**16.53**	0.122	**0.110**
Hapmap58150-rs29020620	6	96724594	**20.75**	7.37	**0.188**	0.096
BTB-00281303	6	111612203	**15.31**	2.36	**0.206**	0.104
ARS-BFGL-NGS-14880	7	53879989	**12.33**	17.75	**0.277**	0.125
ARS-BFGL-NGS-113181	7	62800839	**12.44**	**10.95**	**0.186**	**0.085**
Hapmap44053-BTA-28733	8	6369477	**16.59**	**14.72**	**0.232**	**0.141**
Hapmap31805-BTA-154485	8	36232703	**11.49**	4.12	**0.187**	0.074
Hapmap57000-rs29011304	9	26662	13.22	**19.26**	0.112	**0.109**
BTB-00389006	9	39062436	16.14	**15.30**	0.144	**0.134**
BTA-83317-no-rs	9	40393986	**26.21**	**19.61**	**0.212**	**0.119**
BTA-83528-no-rs	9	41691114	**25.03**	16.16	**0.330**	0.134
BTA-59878-no-rs	9	44095822	28.62	**15.86**	0.140	**0.100**
ARS-BFGL-NGS-52530	9	44230587	**25.18**	**25.13**	**0.275**	**0.224**
ARS-BFGL-NGS-103934	9	44255942	17.70	**18.51**	0.224	**0.138**
BTA-10828-no-rs	9	46600974	24.96	**21.33**	0.109	**0.128**
Hapmap24524-BTA-107865	9	47934344	10.86	**17.83**	0.064	**0.105**
ARS-BFGL-NGS-51043	9	51493578	7.19	**11.78**	0.004	**0.091**
BTB-00391835	9	52160813	**11.19**	5.86	**0.202**	0.101
ARS-BFGL-NGS-114465	9	79835130	7.21	**12.68**	0.032	**0.099**
UA-IFASA-2589	9	82175488	**16.53**	14.78	**0.175**	0.095
ARS-BFGL-NGS-88689	11	29901111	7.51	**14.05**	0.176	**0.171**
ARS-BFGL-NGS-87426	11	30070765	11.64	**14.05**	0.140	**0.143**
ARS-BFGL-NGS-21332	11	30108643	8.57	**14.57**	0.171	**0.159**
ARS-BFGL-NGS-118724	11	30366110	12.31	**11.69**	0.171	**0.145**
Hapmap45971-BTA-102151	11	71170530	15.89	**11.36**	0.071	**0.107**
ARS-BFGL-BAC-12483	13	1310816	9.44	**12.89**	0.185	**0.138**
Hapmap45253-BTA-15908	13	1498184	**11.23**	8.74	**0.250**	0.159
ARS-BFGL-NGS-63777	13	67075815	21.41	**16.44**	0.253	**0.162**
ARS-BFGL-NGS-52851	13	77352863	11.80	**15.82**	0.114	**0.128**
Hapmap29758-BTC-003619	14	5261	**14.75**	11.14	**0.271**	-0.053
Hapmap30383-BTC-005848	14	76704	**45.90**	**32.63**	**0.319**	**-0.148**
BTA-34956-no-rs	14	101474	**21.25**	8.68	**0.258**	-0.126
ARS-BFGL-NGS-57820	14	236533	**72.35**	**54.42**	**0.470**	**-0.162**
ARS-BFGL-NGS-34135	14	260342	**33.86**	17.84	**0.354**	-0.104
ARS-BFGL-NGS-94706	14	281534	**32.66**	16.59	**0.346**	-0.100
ARS-BFGL-NGS-4939	14	443936	**76.53**	**54.67**	**0.497**	**-0.157**
ARS-BFGL-NGS-107379	14	679601	**61.30**	**51.49**	**0.391**	**-0.136**
Hapmap25384-BTC-001997	14	835055	**27.04**	14.45	**0.247**	-0.061
Hapmap24715-BTC-001973	14	856890	**22.96**	11.30	**0.253**	-0.064
BTA-35941-no-rs	14	894253	**25.36**	22.23	**0.375**	-0.076
ARS-BFGL-NGS-101653	14	931163	**20.57**	14.60	**0.322**	-0.094

ARS-BFGL-NGS-26520	14	996983	**20.29**	13.50	**0.361**	-0.058
UA-IFASA-6878	14	1044040	**32.09**	28.23	**0.176**	-0.076
ARS-BFGL-NGS-22866	14	1131951	**20.13**	10.87	**0.307**	-0.098
ARS-BFGL-NGS-103064	14	1193335	**22.91**	7.53	**0.264**	-0.050
ARS-BFGL-NGS-3122	14	1264232	**19.19**	6.75	**0.220**	-0.029
Hapmap25486-BTC-072553	14	1285036	**19.18**	7.04	**0.262**	-0.076
Hapmap30646-BTC-002054	14	1461084	**25.97**	**27.24**	**0.346**	**0.130**
Hapmap30086-BTC-002066	14	1490177	**22.30**	21.27	**0.358**	-0.091
Hapmap30374-BTC-002159	14	1546590	**26.64**	21.65	**0.370**	-0.073
ARS-BFGL-NGS-74378	14	1889209	**18.11**	16.86	**0.236**	-0.059
ARS-BFGL-NGS-117542	14	1913107	**16.54**	12.41	**0.249**	-0.107
ARS-BFGL-NGS-100480	14	2607582	**11.61**	6.51	**0.292**	-0.033
UA-IFASA-5306	14	2711614	**11.40**	11.87	**0.236**	-0.029
Hapmap22692-BTC-068210	14	3018725	**12.60**	13.43	**0.193**	-0.047
Hapmap23302-BTC-052123	14	3099634	**14.23**	18.84	**0.215**	-0.050
UA-IFASA-6329	14	3465238	**10.89**	16.37	**0.231**	-0.036
ARS-BFGL-NGS-110563	14	3799229	**12.33**	14.60	**0.250**	-0.024
Hapmap32262-BTC-066621	14	3834070	**14.68**	9.51	**0.213**	-0.071
Hapmap32236-BTC-049785	14	5139497	12.66	**20.47**	0.142	**0.111**
BTB-01988444	14	56535371	3.79	**10.99**	0.077	**0.143**
ARS-BFGL-NGS-100131	15	21041772	9.65	**10.88**	0.076	**0.145**
BTB-01698088	16	9738423	11.30	**14.27**	0.156	**0.144**
ARS-BFGL-NGS-56645	16	23920210	**15.64**	5.92	**0.199**	0.060
ARS-BFGL-NGS-38023	16	33318455	18.62	**13.72**	0.105	**0.128**
ARS-BFGL-NGS-26559	16	33367687	**11.76**	10.47	**0.216**	0.141
ARS-BFGL-NGS-59645	16	73117625	**16.15**	10.33	**0.235**	0.086
ARS-BFGL-NGS-21141	17	3125444	5.55	**14.51**	0.092	**0.105**
Hapmap58542-rs29018933	17	29430592	4.76	**12.24**	0.071	**0.087**
ARS-BFGL-BAC-33672	20	44108671	10.55	**12.67**	0.235	**0.196**
BTA-17135-no-rs	20	49557519	11.68	**16.41**	0.154	**0.168**
BTB-01251603	20	50418473	8.95	**11.30**	0.178	**0.214**
Hapmap54884-rs29017180	20	50564369	4.84	**12.50**	0.059	**0.087**
ARS-BFGL-BAC-34291	20	54837742	8.44	**14.24**	0.145	**0.177**
Hapmap53927-rs29025287	20	56464296	8.63	**11.17**	0.152	**0.185**
ARS-BFGL-BAC-36842	20	58820614	6.52	**11.22**	0.125	**0.151**
BTA-12959-no-rs	21	10922512	**13.78**	9.40	**0.178**	0.109
ARS-BFGL-NGS-101900	21	30314497	**14.16**	5.32	**0.176**	0.104
ARS-BFGL-NGS-110044	21	30892171	**11.75**	3.37	**0.196**	0.050
ARS-BFGL-NGS-55374	25	28795160	**24.35**	**24.33**	**0.275**	**0.224**
ARS-BFGL-NGS-4066	26	9059815	10.32	**13.50**	0.064	**0.115**
ARS-BFGL-NGS-2464	26	18709176	20.66	**13.07**	0.087	**0.126**
ARS-BFGL-NGS-77668	26	18760372	22.13	**17.22**	0.098	**0.104**
ARS-BFGL-NGS-23064	26	18788121	21.97	**16.74**	0.098	**0.103**
ARS-BFGL-NGS-71584	26	18863914	28.62	**19.66**	0.041	**0.134**
BTA-62184-no-rs	26	20014035	17.96	**11.22**	0.078	**0.083**
BTA-60778-no-rs	26	20090833	21.08	**17.89**	0.100	**0.151**
ARS-BFGL-NGS-25126	26	20165024	14.10	**13.56**	0.049	**0.089**
ARS-BFGL-NGS-116902	26	20191815	14.47	**13.97**	0.063	**0.090**
Hapmap31825-BTA-158647	26	21476707	**13.12**	7.25	**0.174**	0.097
ARS-BFGL-NGS-18603	26	21853286	14.86	**13.52**	0.160	**0.095**
ARS-BFGL-NGS-116481	26	22411701	10.02	**11.08**	0.184	**0.084**
Hapmap24832-BTA-138805	26	22449570	10.14	**11.13**	0.184	**0.084**
BTB-00932332	26	22551770	15.62	**12.20**	0.171	**0.140**
ARS-BFGL-NGS-107403	26	23470277	18.20	**12.99**	0.138	**0.132**
BTB-01622498	28	1436040	3.55	**11.44**	0.130	**0.127**
ARS-BFGL-NGS-43501	28	17344828	14.40	**14.28**	0.099	**0.130**
ARS-BFGL-NGS-118693	28	17493199	10.91	**12.75**	0.071	**0.100**
UA-IFASA-6208	28	27379038	**13.36**	7.50	**0.195**	0.038
BTA-99382-no-rs	28	41568645	**12.46**	12.19	**0.187**	0.074

ln(Fat)

Hapmap38956-BTA-43309	1	98853038	**13.07**	10.74	**0.212**	0.206
BTB-01562245	1	113644592	**11.37**	9.54	**0.306**	0.290
BTA-104132-no-rs	1	113669783	**12.14**	9.85	**0.311**	0.293
ARS-BFGL-BAC-7205	1	120983738	**12.75**	9.11	**0.165**	0.117
ARS-BFGL-NGS-88388	2	13718482	**20.06**	16.60	**0.162**	0.130
ARS-BFGL-NGS-112315	2	40959609	**12.90**	3.87	**0.194**	0.128
ARS-BFGL-NGS-44416	2	48557519	**11.37**	7.58	**0.185**	0.121
BTB-00108243	2	112019423	**17.58**	**22.93**	**0.290**	**0.285**
BTB-01678000	3	6985014	**20.47**	18.76	**0.166**	0.121
BTB-01678060	3	7009487	**19.85**	**20.49**	**0.196**	**0.160**
ARS-BFGL-NGS-5956	3	8558755	**18.50**	**14.08**	**0.195**	**0.147**
ARS-BFGL-NGS-112616	3	8598511	**16.94**	**12.10**	**0.180**	**0.133**
Hapmap50814-BTA-89905	3	41486200	**11.61**	7.40	**0.197**	0.120
INRA-648	3	54347354	**13.24**	**12.30**	**0.309**	**0.288**
Hapmap43441-BTA-103289	3	61621627	17.33	**21.78**	0.141	**0.137**
BTA-68164-no-rs	3	68215262	**12.86**	6.92	**0.199**	0.110
BTB-00131847	3	68241075	**12.93**	7.45	**0.203**	0.119
BTA-54952-no-rs	4	11830564	**11.77**	9.97	**0.184**	0.139
ARS-BFGL-NGS-20815	4	15021946	**16.20**	14.19	**0.171**	0.121
ARS-BFGL-NGS-117196	4	53206872	9.67	**11.42**	0.150	**0.143**
BTB-01252613	5	1851325	**10.90**	7.03	**0.182**	0.150
Hapmap39895-BTA-15668	5	15392995	**10.89**	6.92	**0.189**	0.131
ARS-BFGL-NGS-108617	5	98082173	**15.13**	**11.12**	**0.224**	**0.171**
ARS-BFGL-NGS-95906	5	100351926	**20.56**	**16.92**	**0.192**	**0.133**
Hapmap53294-rs29016908	5	101090418	**39.46**	**25.68**	**0.178**	**0.153**
Hapmap60021-ss46526426	5	101979581	**21.63**	**16.47**	**0.200**	**0.142**
BTB-00270281	6	95770023	**16.41**	9.91	**0.201**	0.123
Hapmap58150-rs29020620	6	96724594	**11.60**	5.12	**0.163**	0.100
BTB-01700063	6	99086447	**21.30**	14.69	**0.237**	0.153
Hapmap53916-rs29021982	6	99581269	**18.02**	**13.96**	**0.196**	**0.150**
Hapmap48078-BTA-77495	6	99827767	**13.14**	12.88	**0.158**	0.116
ARS-BFGL-NGS-14880	7	53879989	**14.54**	13.67	**0.240**	0.155
ARS-BFGL-NGS-113181	7	62800839	**17.30**	17.77	**0.161**	0.122
BTB-02035459	7	63196194	**11.49**	7.58	**0.152**	0.102
BTB-01219396	7	63221359	**11.49**	7.58	**0.152**	0.102
BTA-12616-no-rs	7	64712171	**12.78**	8.69	**0.154**	0.100
ARS-BFGL-NGS-65419	7	66102696	**16.31**	**13.08**	**0.191**	**0.130**
ARS-BFGL-NGS-12863	7	68960712	**16.46**	7.71	**0.215**	0.135
ARS-BFGL-NGS-23091	7	69342623	**13.36**	6.96	**0.227**	0.136
BTB-01222854	7	74587278	**13.33**	9.64	**0.218**	0.151
BTA-23130-no-rs	7	76690557	**11.05**	5.66	**0.174**	0.096
BTB-01321253	7	83625073	**20.03**	20.87	**0.175**	0.126
Hapmap44053-BTA-28733	8	6369477	**15.72**	**11.82**	**0.201**	**0.183**
BTB-01184997	8	36171902	**11.71**	9.07	**0.205**	0.141
Hapmap31805-BTA-154485	8	36232703	**12.94**	9.44	**0.162**	0.110
Hapmap41758-BTA-116042	8	37197678	**11.21**	6.40	**0.224**	0.160
BTB-00384442	9	22407445	**10.97**	7.61	**0.198**	0.127
BTA-83317-no-rs	9	40393986	**18.23**	14.82	**0.183**	0.131
BTA-83528-no-rs	9	41691114	**18.04**	**15.22**	**0.286**	**0.207**
ARS-BFGL-NGS-52530	9	44230587	**13.85**	10.44	**0.238**	0.161
Hapmap34441-BES9_Contig154_536	9	73154820	**12.06**	**11.10**	**0.175**	**0.151**
Hapmap50263-BTA-122214	10	70455224	**11.11**	9.21	**0.271**	0.198
Hapmap30370-BTA-99862	11	14499391	**13.90**	7.21	**0.213**	0.170
Hapmap33349-BTA-127624	12	47348606	10.61	**11.04**	0.176	**0.137**
Hapmap29758-BTC-003619	14	5261	**39.84**	**35.22**	**0.234**	**0.181**
Hapmap30381-BTC-005750	14	50873	**59.74**	**58.91**	**0.274**	**0.229**
Hapmap30383-BTC-005848	14	76704	**64.76**	**61.82**	**0.276**	**0.224**
BTA-34956-no-rs	14	101474	**29.60**	**33.07**	**0.223**	**0.179**
ARS-BFGL-NGS-57820	14	236533	**146.22**	**135.30**	**0.407**	**0.327**

ARS-BFGL-NGS-34135	14	260342	**78.42**	**79.35**	**0.307**	**0.244**
ARS-BFGL-NGS-94706	14	281534	**74.85**	**76.21**	**0.300**	**0.240**
ARS-BFGL-NGS-4939	14	443936	**157.26**	**144.90**	**0.430**	**0.343**
ARS-BFGL-NGS-71749	14	596340	**41.92**	**40.96**	**0.306**	**0.260**
ARS-BFGL-NGS-107379	14	679601	**100.53**	**86.20**	**0.339**	**0.263**
ARS-BFGL-NGS-18365	14	741868	**46.78**	**38.21**	**0.176**	**0.159**
Hapmap30922-BTC-002021	14	763332	**49.33**	**41.41**	**0.181**	**0.175**
Hapmap25384-BTC-001997	14	835055	**64.12**	**62.10**	**0.214**	**0.208**
Hapmap24715-BTC-001973	14	856890	**61.25**	**60.82**	**0.219**	**0.210**
BTA-35941-no-rs	14	894253	**97.77**	**85.15**	**0.324**	**0.255**
ARS-BFGL-NGS-101653	14	931163	**41.53**	**38.61**	**0.279**	**0.240**
ARS-BFGL-NGS-26520	14	996983	**69.06**	**65.29**	**0.313**	**0.274**
UA-IFASA-6878	14	1044040	**35.95**	29.85	**0.152**	0.115
ARS-BFGL-NGS-22866	14	1131951	**44.65**	**45.92**	**0.266**	**0.201**
ARS-BFGL-NGS-103064	14	1193335	**62.36**	**64.75**	**0.229**	**0.242**
ARS-BFGL-NGS-3122	14	1264232	**34.28**	**34.89**	**0.190**	**0.220**
Hapmap25486-BTC-072553	14	1285036	**34.91**	**40.10**	**0.227**	**0.221**
Hapmap30646-BTC-002054	14	1461084	**68.84**	**57.75**	**0.299**	**0.242**
Hapmap30086-BTC-002066	14	1490177	**110.42**	**94.52**	**0.310**	**0.255**
Hapmap30374-BTC-002159	14	1546590	**89.68**	**76.49**	**0.320**	**0.243**
ARS-BFGL-NGS-74378	14	1889209	**48.22**	**36.95**	**0.205**	**0.172**
ARS-BFGL-NGS-117542	14	1913107	**28.04**	**26.03**	**0.215**	**0.189**
UA-IFASA-9288	14	2201869	**48.16**	**32.53**	**0.183**	**0.167**
Hapmap24777-BTC-064977	14	2261622	**13.82**	**12.31**	**0.166**	**0.163**
Hapmap32970-BTC-064990	14	2288509	**32.04**	**27.00**	**0.167**	**0.164**
Hapmap24986-BTC-065021	14	2313594	**32.04**	**27.00**	**0.167**	**0.164**
ARS-BFGL-NGS-22111	14	2347218	18.98	**18.88**	0.122	**0.140**
UA-IFASA-7269	14	2370255	18.98	**18.88**	0.122	**0.140**
Hapmap26072-BTC-065132	14	2391825	23.93	**24.54**	0.144	**0.156**
ARS-BFGL-NGS-113575	14	2484498	**34.94**	**31.97**	**0.178**	**0.164**
ARS-BFGL-NGS-118081	14	2511264	**41.44**	**40.47**	**0.238**	**0.199**
ARS-BFGL-NGS-56327	14	2580413	**60.00**	**53.41**	**0.219**	**0.199**
ARS-BFGL-NGS-100480	14	2607582	**74.83**	**67.79**	**0.253**	**0.223**
UA-IFASA-5306	14	2711614	**47.61**	**32.91**	**0.204**	**0.185**
Hapmap27703-BTC-053907	14	2826072	26.82	**24.14**	0.131	**0.133**
Hapmap22692-BTC-068210	14	3018725	**36.74**	**24.94**	**0.167**	**0.142**
Hapmap23302-BTC-052123	14	3099634	**35.66**	**21.05**	**0.186**	**0.154**
UA-IFASA-6329	14	3465238	**25.62**	**19.44**	**0.200**	**0.159**
ARS-BFGL-NGS-3571	14	3587017	**26.26**	**20.08**	**0.208**	**0.163**
ARS-BFGL-NGS-110563	14	3799229	**25.21**	**17.13**	**0.216**	**0.161**
Hapmap32262-BTC-066621	14	3834070	**13.02**	8.96	**0.185**	0.139
ARS-BFGL-NGS-115947	14	3865963	**33.68**	**21.03**	**0.225**	**0.177**
Hapmap51646-BTA-86764	14	4302230	23.90	**16.80**	0.128	**0.129**
Hapmap30988-BTC-056315	14	4693900	**20.21**	**18.28**	**0.205**	**0.158**
ARS-BFGL-NGS-110894	14	5282437	**14.60**	9.05	**0.197**	0.149
UA-IFASA-6647	14	5808643	**21.04**	13.97	**0.152**	0.111
ARS-BFGL-NGS-102953	14	5867265	**18.86**	10.66	**0.157**	0.114
ARS-BFGL-NGS-16622	15	64781292	**11.60**	9.80	**0.191**	0.153
Hapmap54310-rs29012181	16	8166691	9.54	**10.99**	0.195	**0.138**
ARS-BFGL-NGS-56645	16	23920210	**11.94**	8.17	**0.172**	0.098
ARS-BFGL-NGS-26559	16	33367687	**11.84**	**12.32**	**0.187**	**0.148**
BTA-16056-no-rs	17	20423826	11.69	**11.86**	0.368	**0.345**
ARS-BFGL-NGS-91287	18	10052638	**17.05**	**15.68**	**0.247**	**0.154**
ARS-BFGL-NGS-111247	19	43146804	**14.75**	12.96	**0.158**	0.109
ARS-BFGL-NGS-24479	19	45901285	**11.53**	6.99	**0.188**	0.114
ARS-BFGL-NGS-113693	19	45926259	**12.69**	7.75	**0.188**	0.114
Hapmap48608-BTA-111028	20	52535573	12.23	**16.55**	0.159	**0.144**
BTA-12959-no-rs	21	10922512	**12.62**	9.98	**0.154**	0.108
ARS-BFGL-NGS-101900	21	30314497	**12.91**	7.10	**0.153**	0.096
ARS-BFGL-NGS-55374	25	28795160	**14.21**	10.68	**0.238**	0.161

ARS-BFGL-NGS-39397	26	21166268	**13.43**	**12.13**	**0.163**	**0.147**
Hapmap46411-BTA-15820	26	21404446	**14.57**	**12.21**	**0.197**	**0.179**
Hapmap31825-BTA-158647	26	21476707	**15.83**	**12.66**	**0.151**	**0.143**
ARS-BFGL-NGS-110077	26	21729361	**14.14**	**16.15**	**0.180**	**0.149**
ARS-BFGL-NGS-116481	26	22411701	**22.85**	**23.56**	**0.159**	**0.133**
Hapmap24832-BTA-138805	26	22449570	**23.76**	**24.42**	**0.159**	**0.133**
ARS-BFGL-NGS-6259	26	22492302	**21.29**	**22.06**	**0.159**	**0.133**
ARS-BFGL-NGS-1092	26	24837303	**16.65**	**19.74**	**0.195**	**0.146**
UA-IFASA-4715	26	25330026	16.09	**18.89**	0.128	**0.121**
ARS-BFGL-NGS-38386	26	32836475	7.32	**11.01**	0.134	**0.157**
ARS-BFGL-NGS-105944	26	34196569	**11.54**	8.78	**0.221**	0.146
ARS-BFGL-NGS-53731	26	37801879	13.47	**15.29**	0.131	**0.140**
UA-IFASA-6208	28	27379038	**12.29**	8.03	**0.169**	0.117

ln(Milk)

ARS-BFGL-BAC-13578	1	121811393	**14.00**	**12.21**	**0.197**	**0.168**
ARS-BFGL-NGS-86079	2	19126180	**13.56**	7.17	**0.203**	0.172
Hapmap53232-rs29020795	2	19202356	**12.37**	8.78	**0.177**	0.149
Hapmap60669-rs29018484	2	20687353	**14.57**	10.61	**0.346**	0.238
ARS-BFGL-NGS-75548	4	78246158	**15.29**	8.67	**0.462**	0.309
ARS-BFGL-NGS-115922	5	29921450	10.99	**11.43**	0.278	**0.228**
BTA-75680-no-rs	6	31470687	**11.54**	6.24	**0.161**	0.101
Hapmap54442-rs29025673	6	31579806	**12.50**	7.42	**0.161**	0.101
UA-IFASA-2111	6	85289127	**14.17**	11.85	**0.195**	0.119
Hapmap25708-BTC-043671	6	88263655	**11.20**	10.56	**0.215**	0.109
Hapmap40845-BTA-97263	6	92788189	**11.64**	5.88	**0.203**	0.125
BTB-01428914	6	93850918	**12.73**	7.77	**0.159**	0.092
Hapmap53417-rs29014877	7	106039868	**12.83**	10.48	**0.168**	0.106
ARS-BFGL-NGS-4062	8	5505897	7.21	**11.81**	0.207	**0.159**
Hapmap31053-BTA-111664	9	27739862	**14.86**	10.09	**0.197**	0.139
BTA-59878-no-rs	9	44095822	**11.31**	8.53	**0.165**	0.142
ARS-BFGL-NGS-52530	9	44230587	**12.96**	**12.03**	**0.285**	**0.201**
ARS-BFGL-NGS-38561	9	45373250	8.38	**11.34**	0.140	**0.131**
BTA-10828-no-rs	9	46600974	**16.48**	**17.49**	**0.169**	**0.157**
Hapmap24524-BTA-107865	9	47934344	8.70	**13.36**	0.126	**0.120**
ARS-BFGL-NGS-62628	9	82136926	10.01	**11.28**	0.157	**0.139**
ARS-BFGL-NGS-78549	11	106534689	**12.86**	**11.55**	**0.250**	**0.185**
ARS-BFGL-BAC-12483	13	1310816	14.05	**15.25**	0.157	**0.156**
BTA-15911-no-rs	13	1371524	12.94	**12.38**	0.170	**0.146**
ARS-BFGL-NGS-56157	13	63208626	**13.37**	5.20	**0.172**	0.079
Hapmap54034-rs29026486	13	65183781	**14.01**	4.09	**0.192**	0.076
ARS-BFGL-NGS-103635	13	67816926	**12.80**	4.96	**0.163**	0.076
Hapmap29758-BTC-003619	14	5261	**42.73**	**42.44**	**0.184**	**0.139**
Hapmap30381-BTC-005750	14	50873	**47.35**	**38.59**	**0.295**	**0.235**
Hapmap30383-BTC-005848	14	76704	**132.62**	**116.70**	**0.386**	**0.277**
BTA-34956-no-rs	14	101474	**69.69**	**49.64**	**0.300**	**0.231**
ARS-BFGL-NGS-94706	14	281534	**110.80**	**85.22**	**0.326**	**0.241**
ARS-BFGL-NGS-107379	14	679601	**170.53**	**162.75**	**0.402**	**0.299**
ARS-BFGL-NGS-18365	14	741868	**38.59**	45.89	**0.204**	0.112
Hapmap30922-BTC-002021	14	763332	**28.82**	33.10	**0.188**	0.096
Hapmap25384-BTC-001997	14	835055	**86.77**	**65.63**	**0.304**	**0.183**
Hapmap24715-BTC-001973	14	856890	**77.38**	**57.27**	**0.299**	**0.183**
BTA-35941-no-rs	14	894253	**84.21**	**90.38**	**0.255**	**0.194**
ARS-BFGL-NGS-101653	14	931163	**49.12**	**42.64**	**0.238**	**0.168**
ARS-BFGL-NGS-26520	14	996983	**56.71**	**48.24**	**0.218**	**0.139**
UA-IFASA-6878	14	1044040	**85.46**	**86.53**	**0.265**	**0.190**
ARS-BFGL-NGS-22866	14	1131951	**54.37**	**48.14**	**0.204**	**0.189**
ARS-BFGL-NGS-3122	14	1264232	**55.37**	**35.73**	**0.272**	**0.131**
Hapmap25486-BTC-072553	14	1285036	**53.65**	**31.62**	**0.277**	**0.165**
Hapmap30646-BTC-002054	14	1461084	**63.96**	**69.53**	**0.223**	**0.160**

Hapmap30086-BTC-002066	14	1490177	**80.04**	**85.10**	**0.253**	**0.182**
Hapmap30374-BTC-002159	14	1546590	**86.76**	**91.59**	**0.248**	**0.196**
ARS-BFGL-NGS-74378	14	1889209	**53.11**	**61.21**	**0.276**	**0.191**
ARS-BFGL-NGS-117542	14	1913107	**37.97**	**36.27**	**0.252**	**0.190**
UA-IFASA-9288	14	2201869	**36.57**	42.92	**0.218**	0.122
Hapmap32970-BTC-064990	14	2288509	**19.61**	18.23	**0.171**	0.079
Hapmap24986-BTC-065021	14	2313594	**19.61**	18.23	**0.171**	0.079
Hapmap26527-BTC-005059	14	2418618	**27.21**	**25.01**	**0.186**	**0.130**
ARS-BFGL-NGS-113575	14	2484498	**15.74**	18.23	**0.178**	0.115
ARS-BFGL-NGS-118081	14	2511264	**27.25**	**25.05**	**0.196**	**0.149**
ARS-BFGL-NGS-56327	14	2580413	**38.75**	**39.00**	**0.233**	**0.156**
ARS-BFGL-NGS-100480	14	2607582	**48.59**	**44.93**	**0.252**	**0.165**
ARS-BFGL-NGS-42263	14	2681399	**24.01**	**17.93**	**0.209**	**0.160**
UA-IFASA-5306	14	2711614	**38.68**	**45.11**	**0.233**	**0.145**
ARS-BFGL-NGS-54400	14	2736946	**21.70**	**15.31**	**0.218**	**0.151**
Hapmap22692-BTC-068210	14	3018725	**36.15**	**43.09**	**0.208**	**0.140**
Hapmap23302-BTC-052123	14	3099634	**38.58**	**51.15**	**0.215**	**0.148**
Hapmap25217-BTC-067767	14	3189311	**36.24**	21.15	**0.195**	0.076
UA-IFASA-6329	14	3465238	**26.01**	28.86	**0.170**	0.106
ARS-BFGL-NGS-56339	14	3498808	**16.13**	**20.80**	**0.166**	**0.134**
UA-IFASA-8927	14	3640095	**18.63**	16.26	**0.157**	0.112
Hapmap30091-BTC-005211	14	3940999	**27.46**	21.04	**0.236**	0.119
ARS-BFGL-BAC-24839	14	3993201	**22.93**	22.70	**0.173**	0.106
ARS-BFGL-NGS-112858	14	4956374	**27.34**	35.86	**0.208**	0.133
Hapmap51078-BTA-87682	14	5064062	**16.06**	12.13	**0.202**	0.110
ARS-BFGL-NGS-55227	14	5085415	**21.02**	33.40	**0.171**	0.130
Hapmap32236-BTC-049785	14	5139497	20.69	**34.26**	0.149	**0.126**
ARS-BFGL-BAC-20965	14	5225005	**23.51**	**18.01**	**0.255**	**0.157**
Hapmap33635-BTC-049051	14	5318260	**15.80**	5.33	**0.197**	0.092
Hapmap27091-BTC-048823	14	5356987	**30.86**	26.34	**0.208**	0.098
Hapmap23851-BTC-048718	14	5387835	**29.32**	22.85	**0.244**	0.113
Hapmap32234-BTC-048199	14	5640337	**33.67**	**36.21**	**0.217**	**0.131**
Hapmap26283-BTC-048098	14	5696728	**16.56**	27.75	**0.158**	0.107
Hapmap25716-BTC-047850	14	5937549	**17.19**	**17.52**	**0.208**	**0.123**
Hapmap23799-BTC-047701	14	6044245	**12.10**	11.67	**0.222**	0.108
ARS-BFGL-BAC-8730	14	6252100	**31.45**	21.44	**0.194**	0.106
Hapmap53312-rs29018332	14	60576872	**20.04**	6.97	**0.166**	0.070
Hapmap43128-BTA-105550	15	52541506	**12.39**	6.76	**0.160**	0.088
Hapmap59019-rs29021918	18	41938135	10.34	**16.90**	0.128	**0.148**
Hapmap39811-BTA-122745	20	35432864	10.75	**11.29**	0.170	**0.145**
BTA-50235-no-rs	20	35883921	7.85	**11.52**	0.237	**0.294**
BTA-50402-no-rs	20	36667999	8.05	**14.59**	0.152	**0.160**
Hapmap26466-BTA-160199	20	36746234	8.46	**13.28**	0.163	**0.166**
BTA-50376-no-rs	20	36915967	**15.91**	**20.90**	**0.168**	**0.137**
Hapmap57531-rs29013890	20	36955574	**13.03**	**14.21**	**0.190**	**0.148**
ARS-BFGL-NGS-37182	22	5301596	**11.30**	10.22	**0.282**	0.273
Hapmap43294-BTA-56514	23	32759583	**11.76**	7.34	**0.311**	0.161
ARS-BFGL-NGS-55374	25	28795160	**12.33**	**11.61**	**0.285**	**0.201**
ARS-BFGL-NGS-2127	26	13563198	**13.62**	10.34	**0.163**	0.130
ARS-BFGL-NGS-2464	26	18709176	**12.92**	**10.90**	**0.154**	**0.149**
ARS-BFGL-NGS-77668	26	18760372	19.84	**21.11**	0.139	**0.154**
ARS-BFGL-NGS-23064	26	18788121	19.17	**19.93**	0.139	**0.154**
ARS-BFGL-NGS-71584	26	18863914	19.57	**17.19**	0.119	**0.144**
BTB-00930720	26	21323659	**12.52**	**11.21**	**0.154**	**0.168**
Hapmap31825-BTA-158647	26	21476707	**13.93**	**11.98**	**0.165**	**0.154**
BTB-00931481	26	21631982	21.92	**19.19**	0.165	**0.170**
ARS-BFGL-NGS-18603	26	21853286	14.66	**16.99**	0.145	**0.147**
ARS-BFGL-NGS-114149	26	22137070	5.96	**10.88**	0.109	**0.137**
BTB-00932332	26	22551770	15.99	**15.53**	0.160	**0.184**
ARS-BFGL-NGS-107403	26	23470277	18.82	**18.04**	0.171	**0.194**

BTA-60935-no-rs	26	23985824	17.32	**15.01**	0.142	**0.146**
ARS-BFGL-NGS-119314	26	25634039	12.11	**14.59**	0.136	**0.157**
BTB-00935537	26	26325360	17.75	**14.89**	0.102	**0.135**
Hapmap28763-BTA-162328	26	26472420	9.67	**11.44**	0.111	**0.129**
ARS-BFGL-NGS-109460	27	46280579	**13.48**	2.64	**0.174**	0.083
BTB-02080610	28	19938671	**12.76**	10.74	**0.206**	0.146
Hapmap58649-rs29011010	28	28185115	**15.98**	8.69	**0.162**	0.125
